# Impact of ITH on PRAD patients and feasibility analysis of the positive correlation gene MYLK2 applied to PRAD treatment

**DOI:** 10.3389/fgene.2025.1589259

**Published:** 2025-05-20

**Authors:** Chuanyu Ma, Guandu Li, Xiaohan Song, Xiaochen Qi, Tao Jiang

**Affiliations:** ^1^ Department of Andrology and Sexual Medicine, The Second Hospital of Dalian Medical University, Dalian, China; ^2^ Department of Urology, First Affiliated Hospital of Dalian Medical University, Dalian, China; ^3^ Department of Hepatobiliary Surgery, Dalian Friendship Hospital, Dalian, China

**Keywords:** prostate adenocarcinoma (PRAD), intra-tumour heterogeneity (ITH), prognosis, immunotherapy, MYLK2

## Abstract

**Introduction:**

Prostate adenocarcinoma (PRAD) is an extremely widespread site of urological malignancy and is the second most common male cancer in the world. Currently, research progress in immunotherapy for prostate treatment is slower compared to other tumours, which is mainly considered to be caused by the low rate of immune response in prostate cancer as a cold tumour. Recent studies have shown that intra-tumour heterogeneity (ITH) is an important impediment to PRAD immunotherapy. Therefore, we set out to investigate the feasibility of judging patients’ disease and knowing the clinical treatment based on the level of ITH.

**Methods:**

Clinical information and transcriptome expression matrices of PRAD samples were gained from The Cancer Genome Atlas (TCGA) database. The ITH-score of PRAD samples was evaluated using the DEPTH algorithm. The optimal cut-off value of RiskScore was calculated based on the difference in survival curves, and PRAD patients were classified into high ITH and low ITH groups based on the optimal cut-off value. Genes with expression differences were screened by differential expression gene analyses (DEGs), and 103 positively correlated differentially expressed genes were identified based on these genes as well as the ITH-score. We conducted multivariate Cox regression to sift for prognostically relevant genes to structure an ITH-related prognostic signature. GO and KEGG pathway enrichment analyses were performed on these 103 positively correlated differentially expressed genes, and the proportion and type of tumour-infiltrating immune cells were assessed by TIMER, CIBERSORT, CIBERSORT-ABS, QUANTISEQ, MCPCOUNTER, XCELL and EPIC algorithms in patients. In addition, we calculated the relevance of immunotherapy and predicted various drugs that might be used for treatment and evaluated the predictive power of survival models under multiple machine learning algorithms through the training set TCGA-PRAD versus the validation set PRAD-FR cohort. Based on the upregulated differential gene and ITH-score correlation ranking, combined with the prognostic performance of the gene, we chose MYLK2 as an elite gene for ITH, and performed cellular experiments to validate it by PCR and WB, as well as CCK8, scratch experiments, and transwell experiments on si-MYLK2 PRAD. Finally, we constructed cox regression models as well as random forest survival models based on the expression levels of SYNPO2L, MYLK2, CKM and MYL3.

**Results:**

We found that lowering the ITH-score resulted in better survival outcomes. We identified 20 highly correlated differentially expressed genes by calculating the correlation coefficient (cor>0.3) between them by DEGs as well as ITH-score, and selected four genes with p-value less than 0.05 (SYNPO2L, MYLK2, CKM and MYL3) by combining with cox regression. Survival analysis based on the differential expression grouping of SYNPO2L, MYLK2, CKM and MYL3 suggested significant survival differences. The results of biofunctional pathway enrichment analysis suggested that the PRAD-ITH gene set had significant expression in the Mucsle Contraction pathway. Macroscopic differences in the immune landscape and differences in responsiveness to immunotherapy existed between ITH-H and ITH-L. The results of the CMap data suggested that NU.1025 was the most likely drug to treat PRAD. The results of our machine learning model constructed based on ITH-score suggest that the random survival forest (RSF) model performs well in both the training and validation sets and has the potential to be used as a clinical prediction model. *In vitro* experiments verified that MYLK2 plays an important role in the proliferation and migration of PRAD. Our results suggest that the implementation of therapeutic strategies based on key ITH genes may bring new hope for PRAD patients.

**Discussion:**

Our findings indicate that ITH may be an important biomarker for the prognosis and characterisation of PRAD and that the ITH-related gene MYLK2 may serve as a novel target for the treatment of PRAD patients.

## 1 Introduction

Prostate adenocarcinoma (PRAD) is a highly heterogeneous urological malignancy and the second most common cancer and the sixth leading cause of cancer-related death in men worldwide (Siegel et al., 2024). Radical prostatectomy, brachytherapy and androgen deprivation therapy are the most commonly used treatments for limited PRAD (Herlemann et al., 2024). However, patients with advanced PRAD who have entered an aggressive stage usually lack an effective cure (Adamaki and Zoumpourlis, 2021). As research into the mechanisms of PRAD treatment advances, advance has been made in the current standard therapy strategies, but the mortality rate of patients with advanced PRAD continues to increase every year. Therefore, it is crucial to develop a new biomarker to address the limitations of clinical treatment for patients with advanced PRAD. One of the important biological characteristics of PRAD is its high level of intra-tumour heterogeneity (ITH) (Yadav et al., 2018).ITH refers to the different molecular and phenotypic characteristics of different subpopulations of tumour cells in the same tumour sample (Jamal-Hanjani et al., 2015). Studies have shown that ITH promotes the development of a wide range of malignant tumours as well as invasive metastasis, and is significantly associated with treatment resistance and disease recurrence (Swanton, 2012; Swarbrick et al., 2024). In addition, ITH may reduce the treatment sensitivity of PRAD and become a novel indicator that influences the prognosis of PRAD patients independently of PSA levels (Rhinehart et al., 2024). With the synergistic development of single-cell sequencing technology and machine learning, researchers have devised several algorithms to quantify ITH, including ABSOLUTE (Carter et al., 2012)、PhyloWGS (Deshwar et al., 2015),based on DNA copy number alteration profiling, MATH (Mroz and Rocco, 2013), EXPANDS (Andor et al., 2014),tITH(Park et al., 2016) based on somatic mutation profiling and sITH(Kim et al., 2019) based on splicome measurements. The Deviant Gene Expression Profiling Tumour Heterogeneity (DEPTH) algorithm developed by Li et al. is a novel algorithm for evaluating ITH based on the mRNA level, which has a wider application prospect and a more significant competitive advantage (Li M. et al., 2020).

In this study, we obtained clinical information and transcriptome expression matrices of PRAD patients from the TCGA database and evaluated the ITH-score of PRAD samples based on the DEPTH algorithm. The optimal cut-off value of the RiskScore was determined by survival curve analysis, and PRAD patients were categorized into ITH-H and ITH-L groups based on the optimal cut-off value. We performed survival analyses and screened the top 20 differentially expressed genes with the highest correlation between the high and low ITH groups. In addition, we used multivariate Cox regression to construct prognostic characteristics significantly associated with ITH. Next, we explored the correlation between ITH and the immune microenvironment and immunotherapy, and predicted small molecule drugs that might be used to treat PRAD. Based on the correlation analysis of differentially expressed genes with ITH-score and the prognostic performance of the genes, MYLK2 was screened as an elite gene for ITH. Finally, we confirmed the expression level of MYLK2 in PRAD and verified its regulatory effect on the invasion and metastatic ability of PRAD cells using *in vitro* experiments, thus revealing the relationship between ITH and PRAD. Our findings indicate that ITH can be used as a reliable biomarker for PRAD, providing a new theoretical basis for the prognosis and individualised treatment of PRAD patients.

## 2 Materials and methods

### 2.1 Acquisition of gene expression profiles and somatic mutation data

RNA-seq expression and clinical information of prostate cancer patients were obtained from The Cancer Genome Atlas (TCGA) data (Supplementary Table S1, https://tcga-data.nci.nih.gov/tcga/) (Wang et al., 2016). After excluding patients with incomplete clinicopathological data, 495 PRAD patients were enrolled in this study for subsequent analysis (Supplementary Table S1).

### 2.2 ITH score calculation

DEPTH is a new algorithm to quantify ITH based on mRNA expression profiling, which shows significant advantages over other traditional ITH assessment algorithms. The DEPTH algorithm was used to assess the ITH-score of 495 PRAD samples, and by analysing the differences in the survival curves of different groups, the two groups with the largest differences were selected to determine the optimal cut-off value of the RiskScore, based on which the patients were classified into two groups, ITH-H and ITH-L.

### 2.3 Identification of differentially expressed genes (DEGs) and pathway enrichment analysis

Differentially expressed genes (DEGs) from high and low ITH patients were analysed using the “limma” R software package with the parameters set to |log2Fold change (FC) |> 1, p < 0.05. The DEGs expression profiles of ITH-H and ITH-L were analysed by analysing ITH-H and ITH-L gene expression profiles in the optimal cut-off grouping and combined with ITH-score to identify 103 DEGs with positive correlation with ITH-score. Differences in gene expression profiles under the optimal cut-off grouping and combined with the ITH-score identified 103 DEGs with positive correlation with the ITH-score. 20 highly correlated differentially expressed genes were identified by calculating correlation coefficients (cor >0.3) between the DEGs and the ITH-score. Heatmaps showing the differentially expressed genes were created by the “pheatmap” package and the “org.Hs.eg.db” package was used to annotate the DEGs. Enrichment analysis of DEGs on GO, KEGG and REACTOME pathways Enrichment analysis of DEGs on GO, KEGG and REACTOME paths is performed with the “clusterProfiler” package.

### 2.4 Prognostic analysis of ITH elite genes

Patients were categorised into high ITH or low ITH groups based on the median or best cut-off value. Kaplan-Meier survival analysis was performed to analyse the correlation between the level of ITH and clinical characteristics after combining the ITH-score with information on clinical characteristics of PRAD patients. Progression Free Interva (PFI) under two different subgroups was compared to assess the prognostic value of the different subgroups.

Based on the 20 highly correlated DEGs, we screened these genes for four elite genes associated with prognosis at p < 0.05 after multivariate Cox regression analysis. We used scatter plots to show their correlation with ITH scores. We applied the “survival” R package after obtaining the best cut-off values for these genes based on the difference in survival analyses to plot the survival curves for this typology.

### 2.5 Immune infiltration fit analysis and immune responsiveness analysis

The immune infiltration fitting algorithms are a class of predictive class algorithms that are identical in that they use gene expression data to detect the abundance of cell types, and tissue types in mixed cell populations. The composition of immune cells/tissues in PRAD patients was analysed by 7 algorithms (CIBERSORT, ESTIMATE, MCPcounter, ssGSEA, TIMER, Xcell, EPIC) and visualised by bar charts. Balloon plots were used to show the distribution of immune cells in correlation with the ITH-score in the Xcell algorithm and to compare the infiltration of immune cells between different ITH groups. Afterwards we showed the correlation between the expression levels of 20 elite genes and each immune cell (fraction) obtained in the Xcell algorithm in the form of a heat map. Then, we used the “ggdisterstats” package to generate scatter plots of the two immune cells (fractions) with the strongest correlations to show their correlation with the ITH-score. Based on the TIDE algorithm, we calculated TIDE values for all PRAD samples, which were used to quantify the immunotherapy responsiveness of PRAD samples. Based on the Submap algorithm, we mapped the immunotherapy outcomes of PRAD samples to SKCM samples and calculated the correlation statistics possible for the responsiveness groupings.

### 2.6 CMap algorithm to identify potential drugs

Based on the CMap algorithm, the MYLK2-based CMap scores of the drugs included in the drug database were calculated and ranked according to the score values, and the top five lowest drugs were selected as potential therapeutic drugs for ITH typing. The CMap algorithm provides us with several potential drugs that can reverse the molecular signature of ITH, which are expected to be the reversal drugs for PRAD (Gao et al., 2019).

### 2.7 Modelling ITH prognostic risk

To evaluate their independent prognostic impact on survival, 20 positively correlated differential genes were applied to construct survival models for multiple machine learning combinations. We applied C-index to assess the forecasting capability of the models. Model combinations that constructed models with more than three features were retained and ranked according to the value of C-index.

### 2.8 Statistical analyses

Statistical analyses for bioinformatics were performed using R software (https://www.r-project.org/, version 4.4.1). The analytical and statistical software used for the experiments was ImageJ and Graphpad Prism.

### 2.9 Cell culture

The human prostate cancer cell lines PC-3 and LNCaP clone FGC were purchased from Procell (Wuhan, China); the human normal prostate epithelial cell line RWPE-1 was purchased from Cellverse Bioscience Technology Co. Memorial Institute 1640 medium (RPMI-1640; Wuhan Pricella Biotechnology Co., Ltd.) PC-3 cells were cultured in Ham’s F-12K (Kaighn’s) medium (F-12K; Wuhan Pricella Biotechnology Co., Ltd.). Biotechnology Co. RPMI-1640 medium and F-12K medium were supplemented with 10% foetal bovine serum (FBS; Wuhan Pricella Biotechnology Co., Ltd.), and 1% streptomycin-penicillin (Wuhan Pricella Biotechnology Co., Ltd.) to culture the cells. −1 cells were cultured in Serum-free medium for keratinocytes and Growth factors (RWPE-1 cells dedicated medium; Cellverse Bioscience Technology Co., Ltd.). All these cell lines were cultured at 37°C and 5% CO2.

### 2.10 siRNA transfection

The sequences of MYLK2-specific siRNA (si-MYLK2) and negative control siRNA (si-NC) were obtained from Suzhou Haixing Biosciences Co. The sequences of MYLK2-specific siRNAs were as follows: #1: 5′-CGG​GAA​UGU​CAG​CAG​UGA​ATT-3′, #2: 5′-GGU​GGU​GAA​UUA​UGA​CCA​ATT-3′ and #3: 5′-CAA​CAG​ACA​AGG​CAC​CUA​ATT-3′. GGU​GGU​GAA​UUA​UGA​CCA​ATT-3′ and #3: 5′-CAA​CAG​AGA​CAA​GGC​ACC​UAA​TT-3′. For transfection, PC-3 cells were inoculated in 6-well plates at 50%–60% confluency; 150 pmol siRNA was confluent in 6ul in 6-well plates using GP-transfect-Mate Transfection Reagent (GenePharma, Inc.). 24–48 h post-transfection was used for subsequent assays.

### 2.11 Quantitative real-time reverse transcription-polymerase chain reaction (qRT-PCR) assays

cDNA was synthesised using the TRIGene Plus Total RNA Extraction Reagent and Auxiliary Kit (GenStar, Inc.) and StarScript ProAll-in-one RT Mix with gDNA Remover (GenStar, Inc.). cDNA was analysed for mRNA levels using the 2× RealStar Universal SYBR qPCR Mix Kit (GenStar, Inc.) according to the manufacturer’s instructions. Gene mRNA levels were analysed using the 2× RealStar Universal SYBR qPCR Mix kit (GenStar, Inc.) according to the manufacturer’s instructions. The primer sequences are shown below: MYLK2, forward 5′-GAC​AAG​GCA​CCT​AAA​GGT​CCC-3′, reverse 5′-TTG​GCT​GCT​AGT​TGA​GGG​GTT​G-3'. Calculated using the 2^−ΔΔCt^ method.

### 2.12 Cell proliferation assay

Cell Counting Kit-8 (CCK-8; APExBIO Technology LLC) was used. Cells were inoculated into 96-well plates at a cell density of 2 × 10^3^ cells/well and cultured in 100 μL of cell culture medium containing 10% foetal bovine serum. Before measuring absorbance, 10 μL of CCK-8 Reagent and 100 μL of serum-free medium were added to each well to remove the effect of serum on the CCK-8 Reagent. After incubating the 96-well plate with CCK-8 Reagent for 2 h at 37°C and 5% CO2, the number of viable cells was assessed by measuring the absorbance at 450 nm using an enzyme marker. This was done every 24 h for a total of 72 h. Finally, cell numbers were plotted over a 3-day period using GraphPad Prism 9.1 (GraphPad Software, Inc.) to reflect the rate of cell proliferation.

### 2.13 5-Ethynyl-2′-deoxyuridine (EdU) assay

PC-3 cells were digested and inoculated in 96-well plates at a density of 5 × 103 cells per well. When the cells in the 96-well plate grew to 70%–80%, then 50 μM EdU (Vazyme Biotech, Nanjing, China) was added to each well and incubated for 2 h. The cells were then washed twice with PBS and then fixed with 4% paraformaldehyde for 15 min. Cells were incubated with glycine solution for 5 min at room temperature to neutralize residual fixative. DNA staining was performed by incubating cells with osmolyte for 10 min at room temperature. Add Staining Work Solution containing Azide 488 and incubate cells at room temperature for 30 min away from light. Staining solution containing Hoechst 33342 was added and cells were incubated at room temperature and protected from light for 5 min. Images were obtained using a DS-Qi2 microscope (Nikon, Shanghai, China).

### 2.14 Wound healing tests

For wound healing assays, cells were cultured in 6-well plates and grown to 100% confluence. Cell monolayers were scraped with a 200 μL pipette tip to form wounds. Representative images of cell migration were taken using a light microscope system.

### 2.15 Cell migration and invasion assays

We used the Transwell assay to detect cell migration and invasion as described previously (Yu-Ju Wu et al., 2020). A Transwell chamber containing an 8 μm membrane filter was used (Labselect, Inc.). Serum-free medium with 2 × 10^4^ cells/well was inoculated into the upper chamber, while the lower chamber was filled with medium containing 10% foetal bovine serum. After 48 h of incubation at 37°C, cells in the lower chamber were fixed with 4% paraformaldehyde fixative for 20 min at room temperature and then placed in crystal violet stain (Beyotime; C0121) for 30 min at room temperature. Finally, 3 random fields of view were counted under a light microscope at ×100 magnification. For the invasion assay, Matrigel (Abwbio, Inc.) was pre-coated into the upper chamber for 3 h. Then, cells (5 × 10^4^) were inoculated into the upper chamber in serum-free medium. The remaining experimental steps were the same as for the migration assay.

### 2.16 Western blot assay

Western blot analysis was performed to evaluate the differential expression of MYLK2 protein in tumour and paracancerous tissues. Protein samples were separated using sodium dodecyl sulfate-polyacrylamide gel electrophoresis and transferred to polyvinylidene difluoride (PVDF) membranes. PVDF membranes were closed with 5% skimmed milk in a shaker for 2 h at 37°C and incubated with primary antibody (anti-MYLK2 antibody, 1:2000, 21173-1-AP, Proteintech) at 4°C overnight. After 3 washes in TBST buffer solution for 30 min, the membranes were incubated with HRP-coupled goat anti-rabbit IgG H&L secondary antibody (1:5000, AS014, ABclonal) for 1.5 h. The membranes were then washed three times with TBST buffer solution for 30 min each time. The results were analysed using the Ultra Sensitive ECL Chemiluminescence Kit (SW134-01; Sevenbio). Immunoblotting was quantified using ImageJ software.

## 3 Result

### 3.1 ITH level assessment and prognostic analysis

**FIGURE 1:**
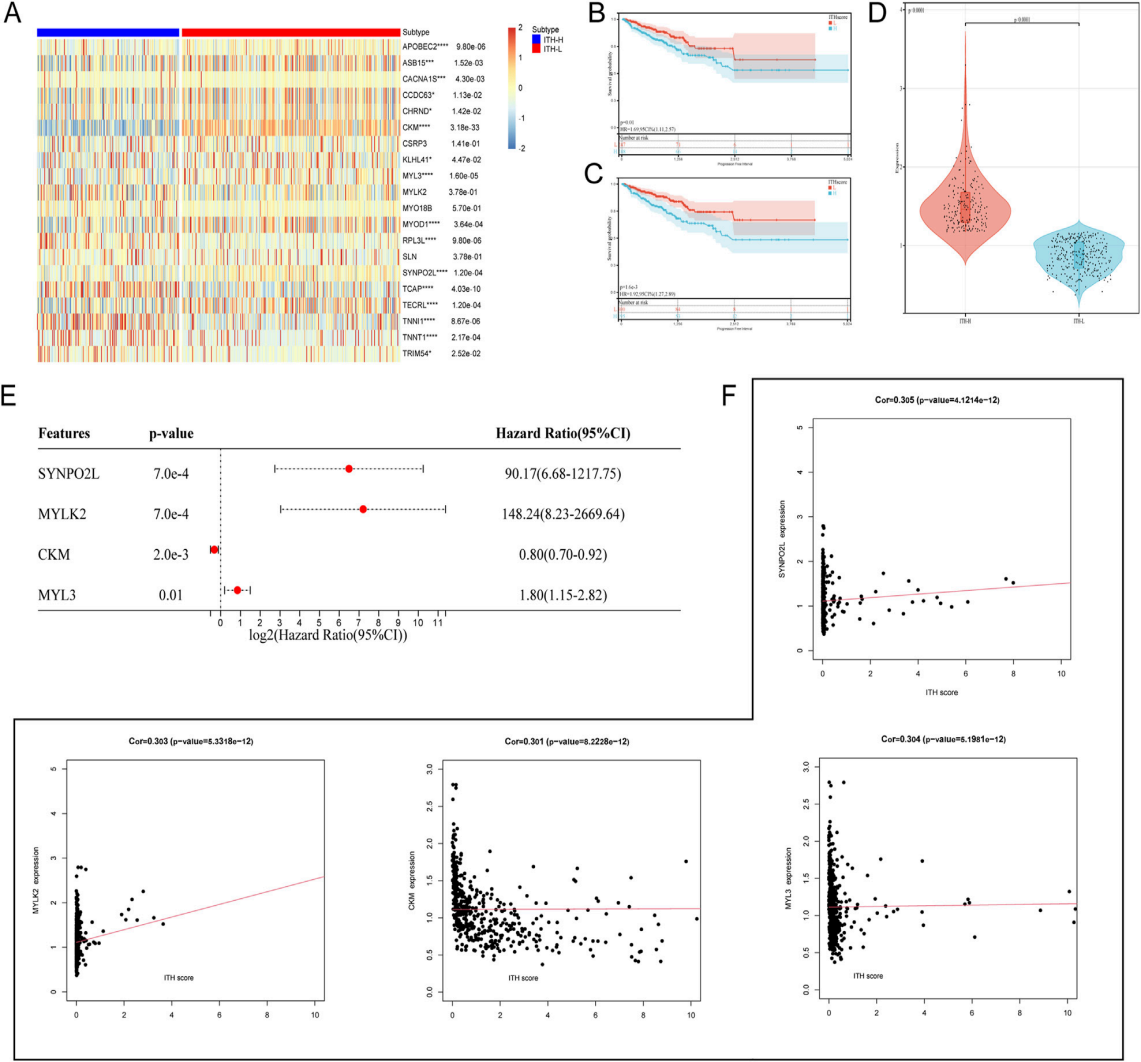
**(A)** Heatmap showing the expression of the 20 DEGs with cor >0.3 in the differential genes of the ITH high and low groups, red: downregulated genes; blue: upregulated genes. Significance levels are indicated by *p < 0.05, **p < 0.01, ***p < 0.005 and ****p < 0.001 **(B)** Progression-free interval (PFI) survival curves grouped at the median of the ITH-score. **(C)** PFI survival curves grouped at the optimal cut-off value of the ITH-score. **(D)** Violin plot demonstrates the data distribution and differences of grouping performed at the best cut-off value of ITH-score. **(E)** Forest plot demonstrating the existence of a significant effect of four genes, SYNPO2L, MYLK2, CKM, and MYL3, on survival outcome in PRAD (HR taken as log2). **(F)** Scatter plot demonstrating the correlation coefficients of ITH-score with SYNPO2L, MYLK2, CKM, and MYL3.

Based on the analysis and collation of mutation data of PRAD samples, the ITH-score of each PRAD sample was calculated using the DEPTH algorithm, and the PRAD samples were classified into two different groups based on the median and the best cut-off value: the high ITH-score group and the low ITH-score group (hereafter referred to as ITH-H and ITH-L). By analysing the differences in gene expression profiles between the ITH-H and ITH-L groups grouped by the best cut-off value and combining with the ITH-score, 103 DEGs with positive correlation with the ITH-score were identified, and the correlation coefficients (cor>0.3) between them were calculated to identify 20 DEGs, and the 20 DEGs between the two groups were visualised on the heatmap (Figure 1A). Kaplan-Meier survival analyses were performed on samples from the median and best cut-off subgroups, respectively, and we found that the lower the ITH score, the better the survival outcome, and that the best cut-off subgroup had a more pronounced differential expression than the median subgroup (Figures 1B, C). Differences were compared and visualised for data from the best cut-off subgroup (Figure 1D). Through multivariate Cox regression, we finally screened four elite genes: synaptotagmin 2-like protein (SYNPO2L), Myosin Light Chain Kinase 2 (MYLK2), Creatine Kinase M-Type (CKM), and Slow Myosin Light Chain 3 (MYL3), in order to construct an ITH-associated prognostic signature (Figure 1E). In addition, we evaluated the correlation analysis of ITH-score with SYNPO2L, MYLK2, CKM, and MYL3 (Figure 1F).

### 3.2 Prognostic analysis of ITH-score related genes

**FIGURE 2:**
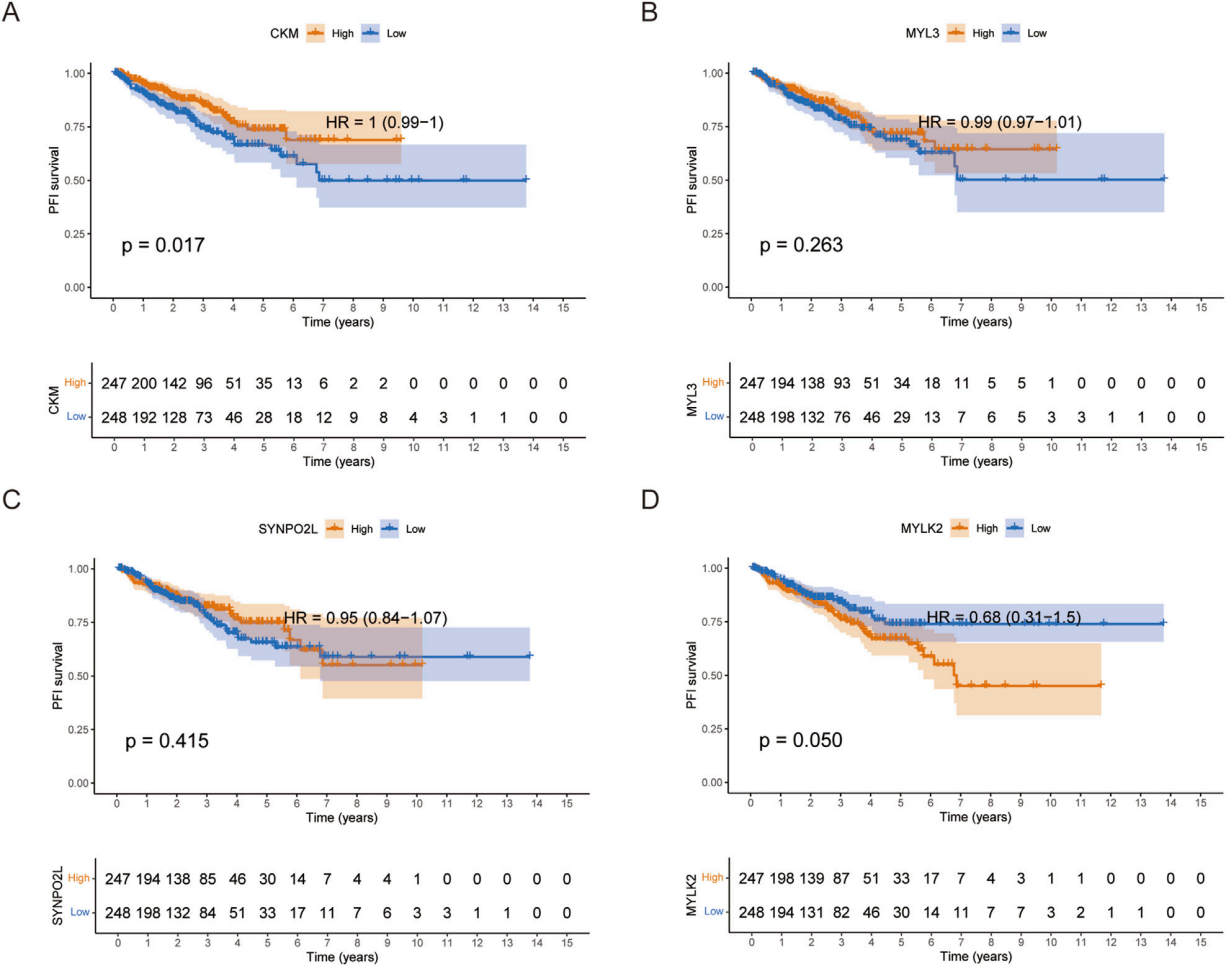
**(A-D)** PFI survival curves grouped by the median expression of CKM, MYL3, SYNPO2L and MYLK2 in PRAD, respectively.

Kaplan-Meier survival analysis based on the median group of differential expression of CKM, MYL3, SYNPO2L and MYLK2 showed that patients with high expression of CKM in Progression Free Interval (PFI) had better survival outcomes, and patients with high expression of MYLK2 had worse survival outcomes. However, there was no statistically significant difference in survival outcomes between patients with high expression of MYL3 and SYNPO2L (Figures 2A–D). Neither patients with high or low expression of these four genes showed significant statistical significance in overall survival (OS), which we hypothesized may be related to the fact that prostate cancer is indolent (Supplementary Figures S1A–D).

### 3.3 ITH pathway analysis

**FIGURE 3:**
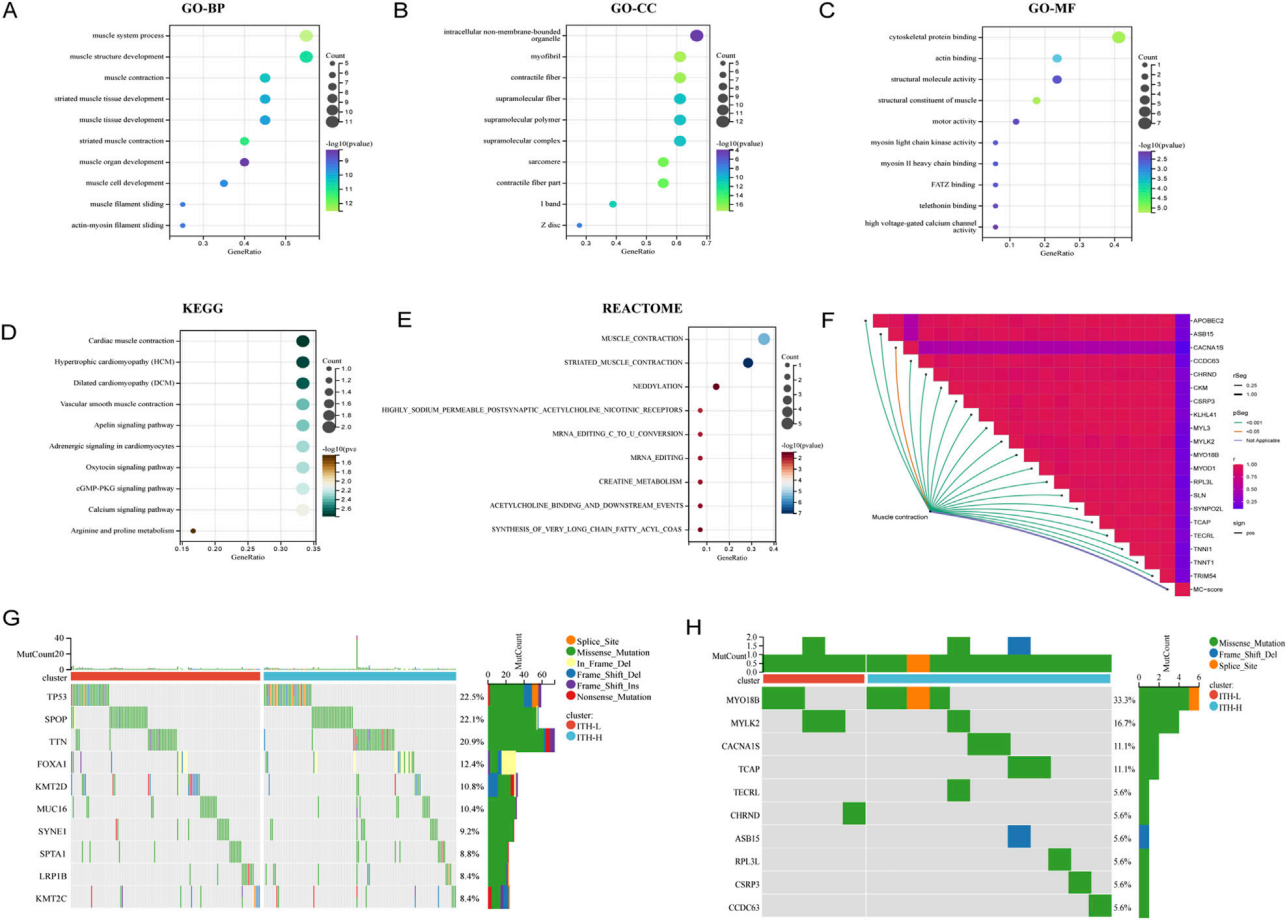
Biological functions and mutation profiles of ITH positively correlated DEGs in PRAD. **(A)** Enrichment results of positively correlated DEGs in PRAD in the BP (Biological Process) section of the GO database. **(B)** Enrichment results of positively correlated DEGs in PRAD in the CC (Cellular Component) section of the GO database. **(C)** Enrichment results of positively correlated DEGs in PRAD in the MF (Molecular Function) section of the GO database. **(D)** Enrichment results of ITH positively correlated DEGs in PRAD in KEGG database. **(E)** Enrichment results of ITH positively correlated DEGs in PRAD in REACTOME database. The size of each bubble indicates the degree of enrichment, and its position indicates the importance of the disease (pathway) affected by the genetic change. **(F)** Correlation connectivity plots demonstrating the correlation between the Mucsle Contraction pathway enriched in multiple databases and all the ITH positively correlated genes. **(G)** Mutation spectrum of TOP10 genes with high mutation rates in PRAD patients under ITH-H/ITH-L subgroup. **(H)** Mutation spectrum of TOP10 mutation rates of all ITH positively correlated genes in PRAD patients under ITH-H/ITH-L subgroup.

Further GO, KEGG and REACTOME enrichment analyses of all the DEGs positively correlated with ITH showed that the genes closely and positively correlated with ITH-score were mainly involved in biological activities such as Cardiac muscle contraction, Mucsle Contraction, muscle system process and muscle structure development, among other biological activities (Figures 3A–E). In addition, the results showed that Mucsle Contraction was significantly expressed in all three databases. Therefore, we demonstrated the correlation between Mucsle Contraction and all ITH positively correlated genes with correlation linkage plots. The results showed that Mucsle Contraction was highly correlated with SYNPO2L, MYLK2, CKM, MYL3, but weakly correlated with CACNA1S (Figure 3F) (Hu et al., 2021). In PRAD patients, a large number of gene mutations were observed between the high and low ITH groups, including TP53, SPOP, TTN, FOXA1, KMT2D, MUC16, SYNE1, SPTA1, LRP1B, and KMT2C, and in addition, we observed that the mutation rate of MYLK2 was located in the second place of the positively correlated genes in ITH (Figures 3G, H). We demonstrated the distribution of differences between ITH-score levels and T-levels, N-levels, M-levels, gleason scores, and age in PRAD patients (Supplementary Figures S2A–E), and more comprehensively showed significant correlations between the elite gene MYLK2 and fustat indicators and all the clinical information mentioned above, as well as the ITH-score, in PRAD patients by heatmaps (Supplementary Figure S2F).

### 3.4 Correlations between ITH-score and immunotherapy target genes and immune cell infiltration

**FIGURE 4:**
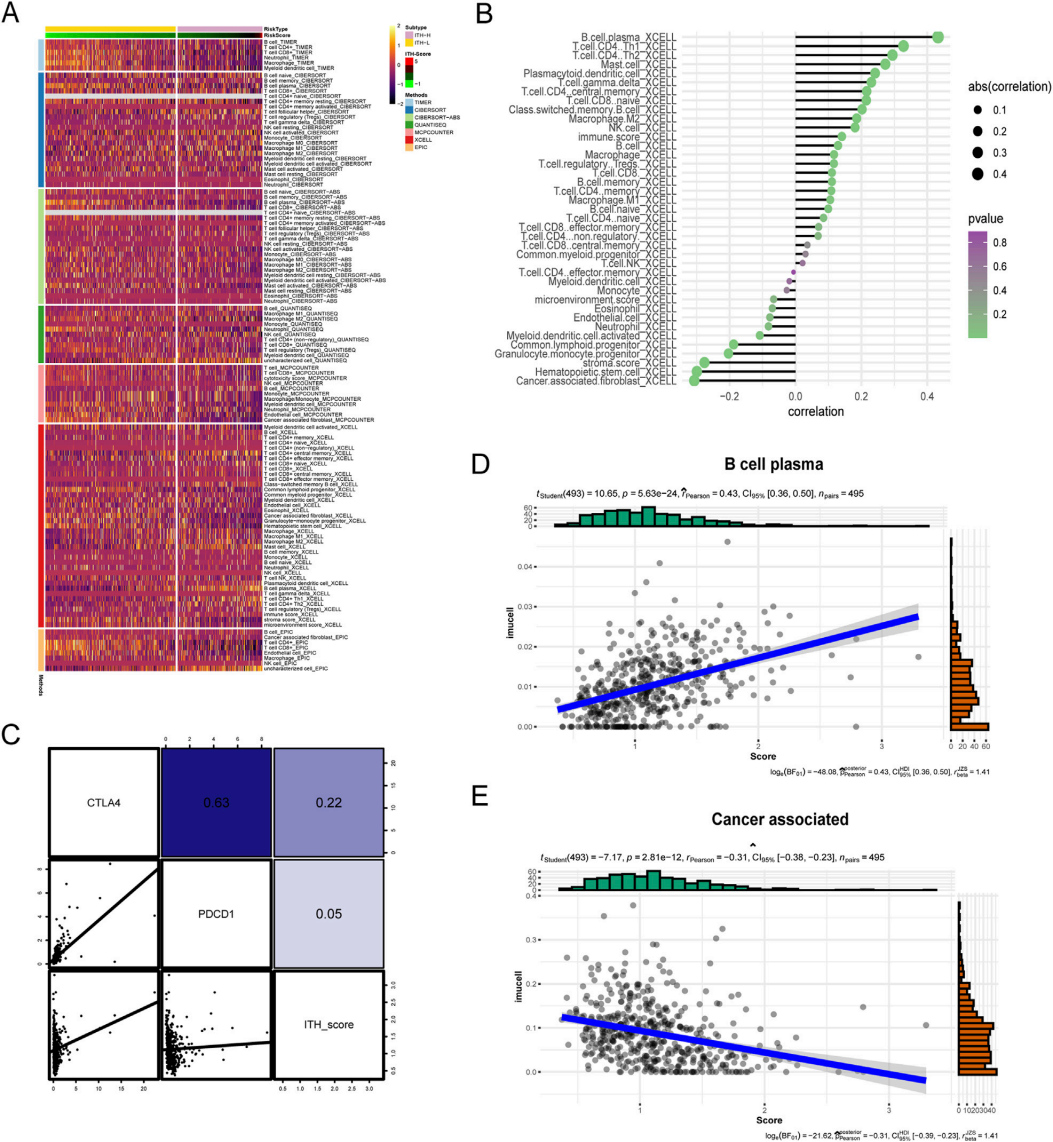
**(A)** Immunoheatmap demonstrating the immune landscape under ITH-H and ITH-L grouping, with different coloured regions representing different immune infiltration algorithms (TIMER, CIBERSORT, CIBERSORT− ABS, QUANTISEQ, MCPCOUNTER, XCELL, and EPIC). **(B)** Balloon plot examining the ITH- correlation between scores and immune cell infiltration. Bubble sizes indicate correlation sizes from 0.1 to 0.4, and colours indicate p-values from 0 to 1. **(C)** Correlation analysis between ITH-score and gene expression of CTLA-4 as well as PD-L1 therapeutic targets. **(D)** Scatterplot demonstrating the correlation between B cell plasma and ITH-score. **(E)** Scatterplot demonstrating the correlation between Cancer associated with the ITH-score.

We comprehensively analysed the macroscopic immune cell infiltration of ITH-score on PRAD by different immune infiltration algorithms (Figure 4A), and the lower the ITH-score, the higher the degree of immune infiltration in TIMER algorithm, CIBERSORT− ABS algorithm, MCPCOUNTER algorithm and EPIC algorithm. The results showed that ITH-L was more inclined to immunothermal tumours and ITH-H was more inclined to immunocold tumours. Therefore, we calculated the correlation between the 20 DEGs identified earlier as highly relevant for ITH by the CIBERSORT algorithm and the TIMER algorithm to fit the abundance of immune cells (components), and the results showed a significant negative correlation between most of the immune infiltration components and the ITH score (Supplementary Figure S3). However, the results calculated by the Xcell algorithm showed a significant positive correlation between B cells and T cells and the ITH score (Figure 4B). In addition, We validated the correlation between ITH score and key immune checkpoint genes in different cancer types (Supplementary Figure S4). Subsequently, we verified the correlation between ITH-score and immune checkpoint-related genes PDCD1 and CTLA4 in PRAD patients, and the results showed a positive correlation between ITH-score and PDCD1 and CTLA4 levels (Figure 4C). We selected the immune factor B cell plasma, which had a high positive correlation, and the immune function Cancer associated, which had a high negative correlation, for correlation analysis, and the results showed that they showed the same trend of correlation and bubble plots with ITH-score (Figures 4D, E).

### 3.5 Interrelationship between ITH-score and immunotherapy

**FIGURE 5:**
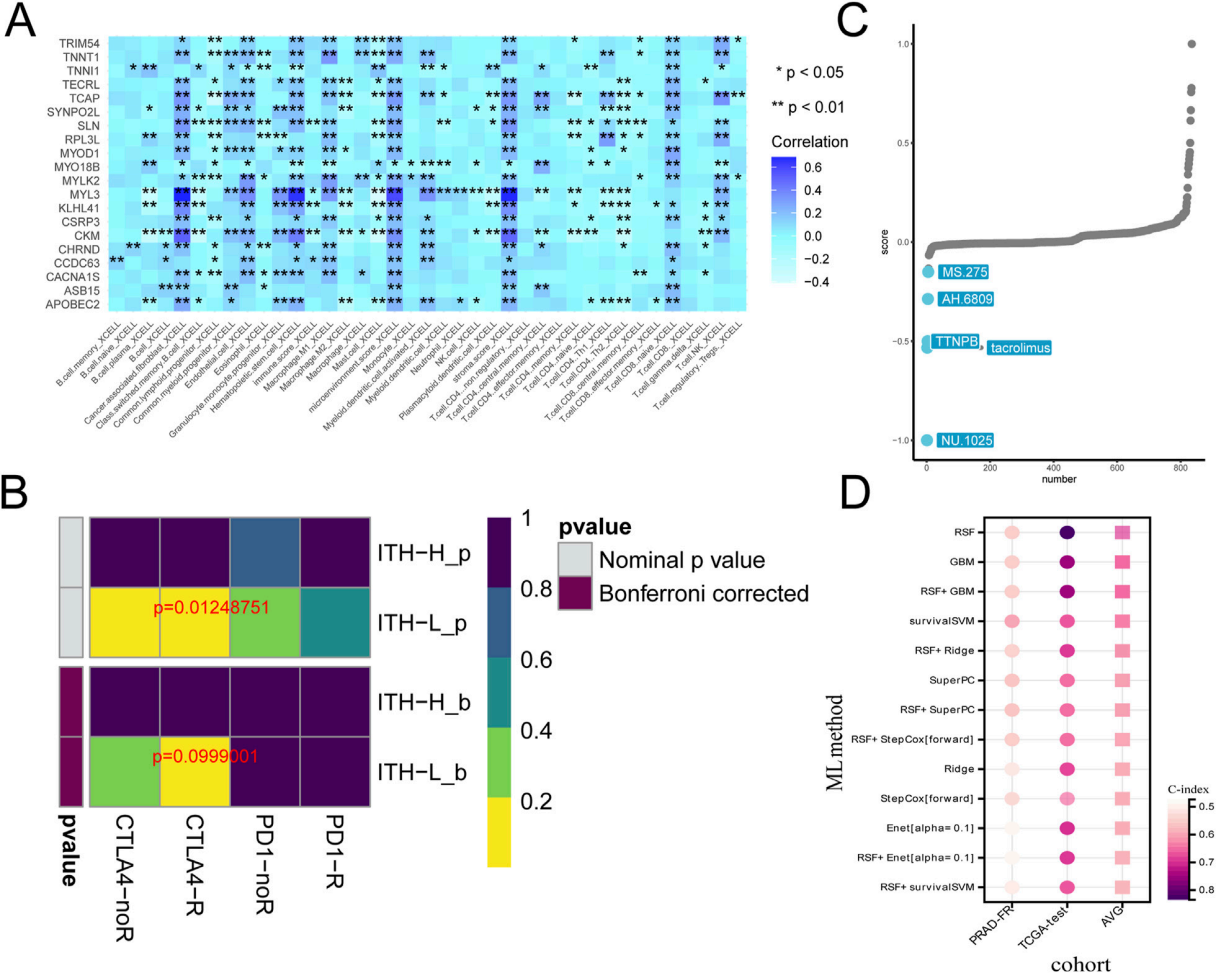
**(A)** Heatmap demonstrating the correlation between the 20 genes expressed between high and low ITH subgroups and the individual immune infiltration components in the X-CELL algorithm. **(B)** Heatmap demonstrating the prediction of p-values obtained by comparing samples in the ITH-H/ITH-L subgroups by using PD-1 and CTLA4 treatments as well as comparative results of p-values after Bonferroni correction. **(C)** CMap analysis plot demonstrating the feasibility of various pharmacological treatments for PRAD. **(D)** Heatmap demonstrating the predictive power of survival models with multiple machine learning algorithms.

In addition to targeted drugs, immunotherapy is gradually gaining widespread attention in cancer treatment. However, although immunotherapy is the main treatment modality for a variety of common tumours, it has not yet become a routine option for PRAD patients (Venturini and Drake, 2019). To examine the regulatory role of ITH in PRAD immunotherapy, we calculated the relationship between 20 DEGs and each immune infiltration component using the X-CELL algorithm. The results showed that MYLK2 had a high correlation with most immune infiltration components, and the highest correlation was between MYL3 and immune infiltration components (Figure 5A). We calculated the correlation between immunosuppressants and PRAD under the ITH-H/ITH-L subgroups, and the heatmap showed statistically significant responses to immune checkpoint CTLA-4 treatment in both the pre-Bonferroni calibration (p = 0.01248751) as well as in the post-calibration (p = 0.0999001) ITH-L group (Figure 5B). In addition, we used the CMap algorithm to predict small molecule drugs that may have an effect on PRAD (Yang et al., 2022). The results showed that NU.1025, tacrolimus, TTNPB, AH.6809, and MS.275 were the top five potential drugs for treating PRAD patients under the ITH-score subgroup (Figure 5C). Subsequently, we predicted survival models for 20 ITH positively correlated differential genes using various machine learning algorithms and applied C-index to assess the forecasting capability of the models. The outcomes indicated that random survival forest (RSF) had the best predictive ability (Figure 5D).

### 3.6 Silencing of ITH-related gene MYLK2 inhibits PC-3 cell invasive metastasis and promotes cell death

**FIGURE 6:**
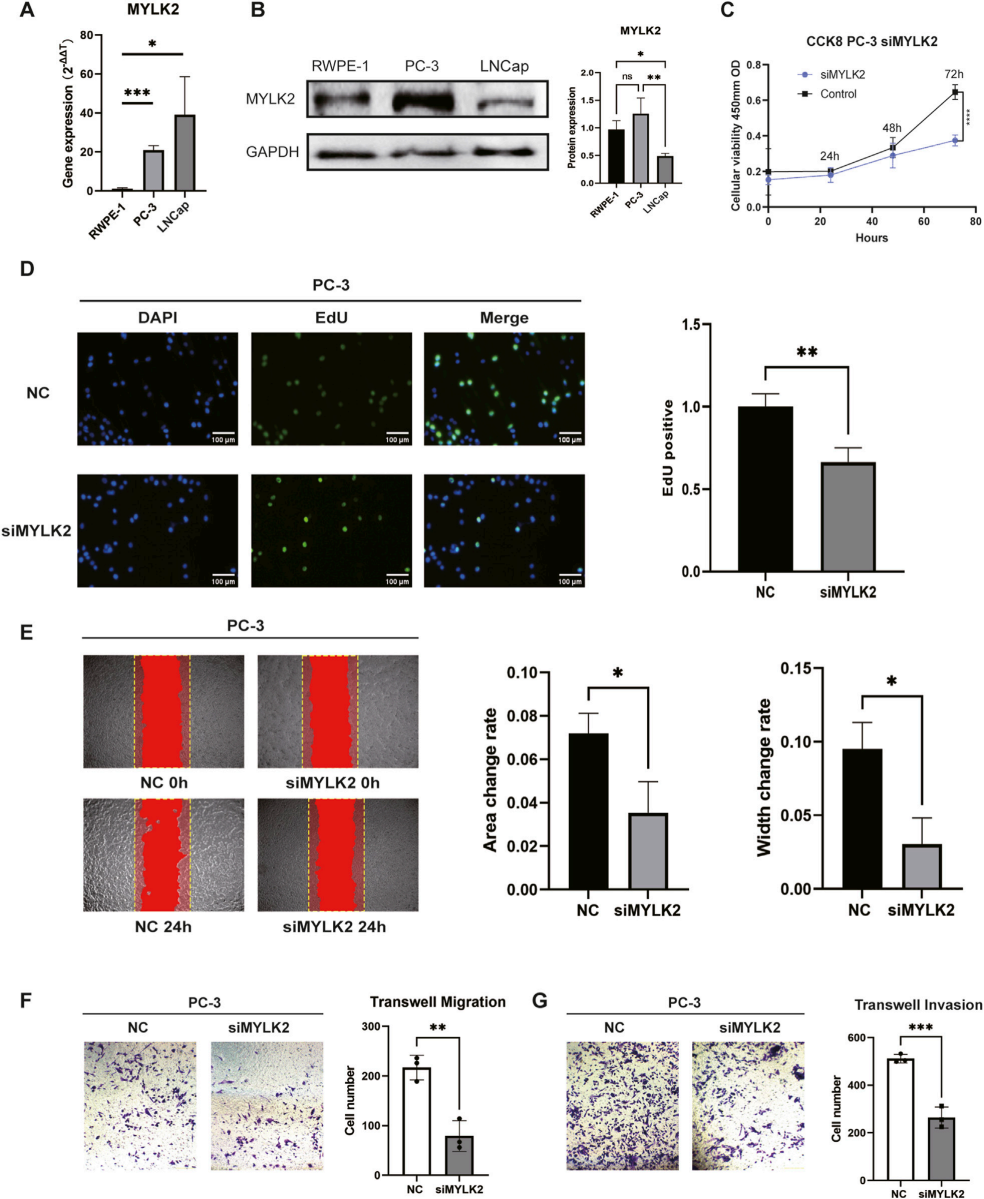
**(A)** q-PCR showing the comparison of mRNA expression of MYLK2 in PC-3 and LNCap cells with RWPE-1 cells. **(B)** WB showing the comparison of protein expression of MYLK2 in PC-3 and LNCap cells with RWPE-1 cells. **(C)** CCK-8 assay showing the proliferative viability of the cells after knocking down MYLK2 compared to the non-knockdown down group in comparison. **(D)** Cell proliferation is measured by the EdU assay. **(E)** Wound healing assay showing the migratory ability of PC-3 cells treated with si-NC or si-MYLK2. The comparison of area change and width change between the two groups were statistically significant. **(F)** Transwell migration assay showing the migration ability PC-3 cells treated with si-NC or si-MYLK2. **(G)** Transwell invasion assay showing the invasion ability of PC-3 cells treated with si-NC or si-MYLK2. Data are expressed as SD ± mean. *P < 0.05, **P < 0.01, ***P < 0.001.

To assess the biological role of MYLK2 in PRAD, small interfering RNAs (siRNAs) specifically targeting MYLK2 binding were designed. MYLK2 RNA expression was validated in several PRAD cell lines. qPCR as well as Western blotting (WB) results showed that MYLK2 expression was relatively high in PC-3 and LNCap cells compared to the normal prostate cancer cell line RWPE-1 (Figures 6A, B). Protein and RNA changes of MYLK2 were detected by WB and qPCR after transfection of PC-3 cells with MYLK2 siRNA for 24–48 h. The results showed that MYLK2 was successfully knocked down (Supplementary Figures S5, 6). CCK-8 and EdU assay demonstrated that downregulation of MYLK2 inhibited the proliferative activity of PC-3 cells as compared to the si-NC group (Figures 6C, D). Wound healing assay and Transwell migration assay showed that silencing of MYLK2 significantly inhibited the migration of PC-3 cells (Figures 6E, F). Transwell invasion assay assay showed that the invasive ability of PC-3 cells was significantly decreased after knockdown of MYLK2 (Figure 6G). These results suggest that the MYLK2 gene is a key therapeutic target for PRAD associated with ITH (Supplementary Table S2).

### 3.7 Machine learning based prognostic judgement for PRAD patients

**FIGURE 7:**
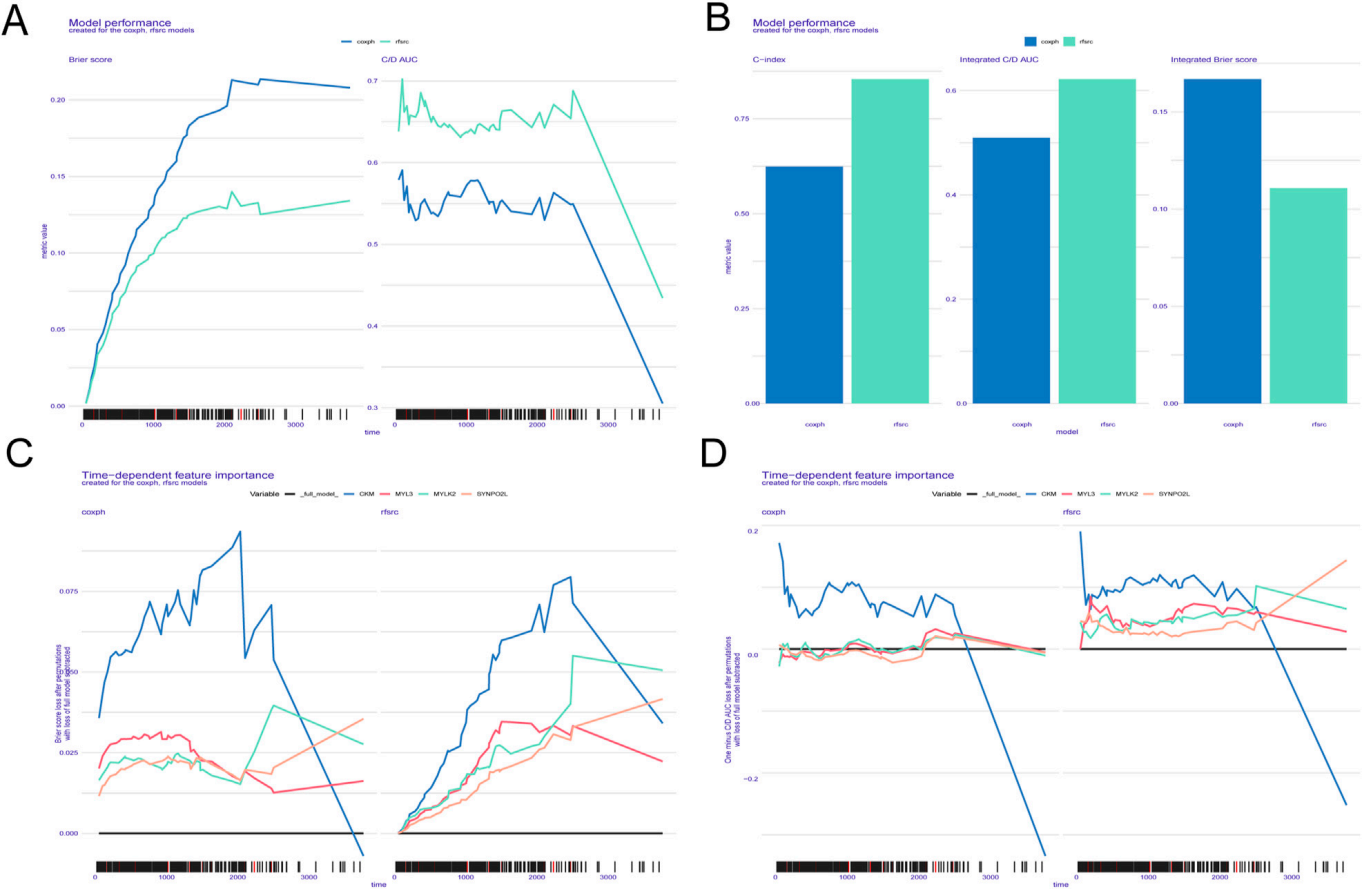
**(A-D)** ITH scores obtained based on the SURVEX package, constructing both the random forest model and the COX regression model of the MOMC‐VM and combining it with machine learning.

According to the Brier score and the C/D AUC index based on survival time, it can be found that the Brier score value of RSF is always lower than that of Cox proportional hazards (CPH), while the C/D AUC value is always higher than that of CPH, which suggests that RSF’s predictive power is better than CPH, and the C-index results are consistent with the previous two results (Figures 7A, B). The line graphs of the importance of time-dependent features showed that the Brier score and the C/D AUC index based on survival time of MYLK2 were greater in RSF than in CPH, which indicated that the importance of MYLK2 was greater in RSF, which had superior predictive ability, than in CPH, and that its importance first increased and then decreased with time. (Figures 7C, D).

## 4 Discussion

Prostate cancer is one of the most common malignancies among men in the United States and ranks as the second leading cause of cancer-related deaths, accounting for 19% of new cancer cases and 9% of cancer deaths (Siegel et al., 2024). The clinical management of prostate adenocarcinoma (PRAD) remains challenging, largely due to its pronounced heterogeneity and strong metastatic potential (Yadav et al., 2018). Intra-tumour heterogeneity (ITH) drives tumour evolution and biological diversity, posing significant obstacles to the diagnosis, prognosis, and treatment of PRAD (McGranahan and Swanton, 2017; Yadav et al., 2018). The critical role of ITH in tumour progression has been further underscored by a large-scale study involving whole-genome sequencing of 2,658 cancer samples across 38 cancer types (Dentro et al., 2021). Although the contribution of ITH to PRAD development is well recognised, its specific impact on patient outcomes and therapeutic strategies remains insufficiently understood. In this study, we assessed the ITH-scores of 495 PRAD samples using the DEPTH algorithm, determined the optimal cut-off value based on survival curve analysis, and stratified patients into high- and low-ITH groups. Differential expression analysis revealed 103 differentially expressed genes (DEGs) associated with ITH. Patients in the high-ITH group exhibited significantly worse progression-free intervals (PFI) compared to those in the low-ITH group. We subsequently constructed ITH-related prognostic models, demonstrating that ITH serves as an independent predictor of PFI in PRAD. Further analysis identified 20 DEGs strongly correlated with ITH-scores (correlation coefficient >0.3), from which four key genes (SYNPO2L, MYLK2, CKM, and MYL3) were selected through multivariate Cox regression (p < 0.05). In addition, we investigated the relationship between ITH and the immune microenvironment, evaluated potential responsiveness to immunotherapy, and predicted small-molecule drugs for PRAD treatment. Among the DEGs, MYLK2 emerged as a particularly significant gene, showing strong correlation with ITH-scores and prognostic relevance.

In this study, our prognostic hypercorrelated genes were SYNPO2L, MYL3, CKM and MYLK2. These four key prognostic genes have been reported to be closely associated with tumour development and invasion and metastasis. SYNPO2L is located in myocyte ganglia and is involved in cardiac morphogenesis (Yamada et al., 2024). SYNPO2L promotes COL10A1 secretion and infiltration of tumour-associated fibroblasts, thereby promoting epithelial-mesenchymal transition (EMT) in tumour cells, making them more likely to develop distant metastases (Wu et al., 2024). MYL3 is an important member of the slow muscle fibres of skeletal muscle, and its expression directly determines the contractile properties of the muscle fibres (Geiger et al., 2024). MYL3 not only acts as a slow muscle fibre, but also serves as a biomarker of poor prognosis in squamous cell carcinoma of the head and neck markers (Li et al., 2023).CKM is an M-type creatine kinase involved in catalysing the transfer of phosphate groups between ATP and phosphocreatine, which in turn affects energy metabolism in the heart (Bertero and Maack, 2018). In heart failure, acetylation may lead to a decrease in CKM activity, thereby exacerbating high-energy phosphorylation (Walker et al., 2021). Recent studies have identified CKM as a possible biomarker for the diagnosis, prognosis and treatment of PRAD (Huang et al., 2024). The MYLK family consists of MYLK, MYLK2, MYLK3, and MYLK4. It has been demonstrated that MYLK influences the progression of hepatocellular carcinomas by catalysing the phosphorylation of myosin light chain (MLC) (Lin et al., 2018). In addition, MYLK promotes PRAD cell invasion and metastasis by regulating miR-29a expression (Dai et al., 2018). MYLK4 accelerates tumour progression through activation of epidermal growth factor receptor signalling in osteosarcoma (Yang et al., 2021). MYLK2 also modulates MLC phosphorylation, which affects the contractile function of smooth, skeletal and cardiac muscle (Stull et al., 2011). However, the role of MYLK2 in cancer progression has not been clarified. Given the role of the MYLK family family in tumour progression, we hypothesised that MYLK2 may play a key role in the development of PRAD and may serve as a marker of worsening prognosis in PRAD. We confirmed that MYLK2 was highly expressed in PRAD by qPCR of normal prostate epithelial cell line RWPE-1 as well as prostate cancer cell lines PC-3 and LNCaP clone FGC, and Western blotting (WB). The effects of MYLK2 on PRAD cell proliferation and invasive metastasis were explored by CCK-8, wound healing and Transwell assays. The results suggest that MYLK2 may become a new biomarker and provide new insights related to ITH for mechanistic studies of PRAD.

The tumour microenvironment (TME) consists of tumour cells, multiple immune cell types, cancer-associated fibroblasts and stromal cells (de Visser and Joyce, 2023). With the progress of TME research, the key role of multiple immune cells in the development, invasion metastasis, and immune resistance of PRAD has received much attention (Lautert-Dutra et al., 2024). However, PRAD has been shown to be an immunocold tumour in most studies, so future research directions could explore how to transform it into an immunothermal tumour for better therapeutic effects (Zheng et al., 2021). Therefore, infiltrating immune cells may be a potential therapeutic target. In this study, we evaluated the types and proportions of infiltrating immune cells in the ITH-H and ITH-L groups by TIMER, CIBERSORT, CIBERSORT− ABS, QUANTISEQ, MCPCOUNTER, XCELL and EPIC algorithms. Our results showed that the ITH-L group was more enriched in immune cells compared to the ITH-H group. In addition, we estimated the correlation between the ITH-score and the abundance of immune cells (fractions) under the Xcell algorithm based on the highly correlated DEGs of ITH. Our results showed that ITH-H correlated more strongly with stromal scores, whereas the ITH-L group correlated more strongly with immune scores. This may explain that the ITH-L group had a better prognosis. Subsequently, we used the X-CELL algorithm to calculate the relationship between ITH highly correlated DEGs and each immune infiltration component. The results showed that MYLK2 was highly correlated with most of the immune infiltration components. This suggests that MYLK2 may serve as a new immunotherapeutic target to improve the prognosis of PRAD patients. In addition, we calculated the correlation of ITH-score with immune checkpoint-related genes as well as immunosuppressants in PRAD patients, and the outcomes indicate that ITH-score was positively correlated with the levels of PDCD1 and CTLA4, and patients in the ITH-L group were more responsive to CTLA-4. This suggests that ITH-score affects the efficacy of immunotherapy, and CTLA-4 may have reference significance for the treatment of ITH-L group. In conclusion, ITH may be involved in the tumour microenvironment, thus affecting tumour development.

We predicted a number of potential small molecule drugs for the treatment of patients with PRAD based on the high relevance of ITH DEGs. NU.1025 is a poly ADP ribose polymerase (PARP) inhibitor that induces apoptosis in breast cancer cells by enhancing cytotoxicity through inhibition of DNA repair (Keung et al., 2020). Tacrolimus binds to FK506 binding protein (FKBP) to form a complex that inhibits the proliferation of colorectal, lung, and liver cancer cells (Hanaki and Shimada, 2023). The RAR agonist TTNPB induces granulocyte differentiation and apoptosis in acute promyelocytic leukaemia (Gianni et al., 2000). AH.6809 inhibits the proliferation and invasion of breast cancer cells by silencing the EP2 receptor and is a potential therapeutic approach for the treatment of metastatic breast cancer (Cheuk et al., 2015). The histone deacetylase (HDAC) inhibitor MS.275 (Entinostat), which is highly specific for class 1 HDACs, selectively induces apoptosis in cancer cells by combining with tetrandrine, but normal cells are unaffected (Li H. et al., 2020). In addition, we constructed a variety of machine learning models based on 20 ITH positively correlated differential genes, and the outcomes suggested that the RSF model had the best forecasting ability and had the potential to be used as a clinical prediction model. Subsequently, we determined the prognostic impact of MYLK2 on PRAD patients based on machine learning, and the results showed that MYLK2 was more important than CPH in RSF, and its importance first increased and then decreased with time, which we hypothesised might be related to late gene mutations in PRAD patients. Our results suggest that the implementation of a therapeutic strategy based on the key ITH gene MYLK2 may bring new hope for PRAD patients.

Limitations of this study need to be noted. Firstly, we constructed and validated these characteristics based only on retrospective data from the TCGA database, and future multicenter and large-scale prospective studies are still needed to further assess their clinical value. Second, we only performed *in vitro* experiments and further *in vivo* studies are needed to explore the role of MYLK2 in PRAD patients.

## Data Availability

The original contributions presented in the study are included in the article/Supplementary Material, further inquiries can be directed to the corresponding authors. The RNA‐seq of TCGA‐PRAD cohort in this study was downloaded from TCGA database (https://www.cancer.gov/ccg/research/genome-sequencing/tcga). This public database allows researchers to download and analyse public datasets for scientific purposes.

## Abbreviations

PRAD, Prostate adenocarcinoma; ITH, Intra-tumour heterogeneity; PFI, Progression Free Interval; REACTOM, Reactom pathway database; GO, Gene ontology; KEGG, Kyoto encyclopedia of genes and genomes; CCK-8, Cell Counting Kit-8; EdU, 5-Ethynyl-2′-deoxyuridine; DEGs, differentially expressed genes; MYLK2, Myosin Light Chain Kinase 2; MYL3, Myosin Light Chain 3; CKM, Creatine Kinase, M-Type; SYNPO2L, Synaptopodin 2 Like; PD-1, Programmed cell death protein 1; CTLA-4, Cytotoxic T lymphocyte-associated antigen-4; CPH, Cox proportional hazards; RSF, random survival forest; TME, tumor microenvironment
