# Supplementary figures and images for: Impact of ITH on PRAD patients and feasibility analysis of the positive correlation gene MYLK2 applied to PRAD treatment
Source: Front Genet. 2025 May 20;16:1589259. doi: 10.3389/fgene.2025.1589259 (PMC12130005; doi:10.3389/fgene.2025.1589259)

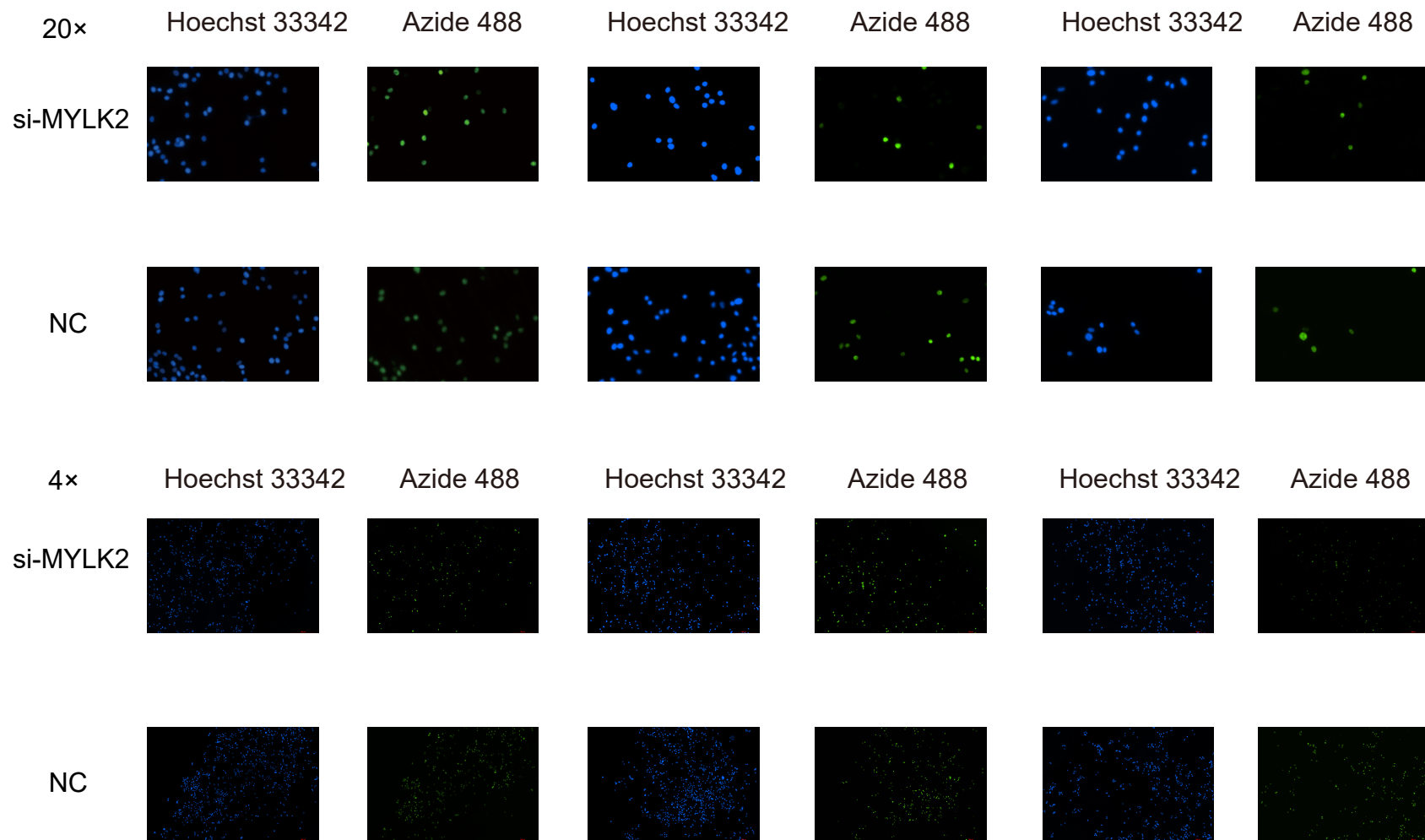

Supplement: Supplementary file 1 [file DataSheet2.pdf]

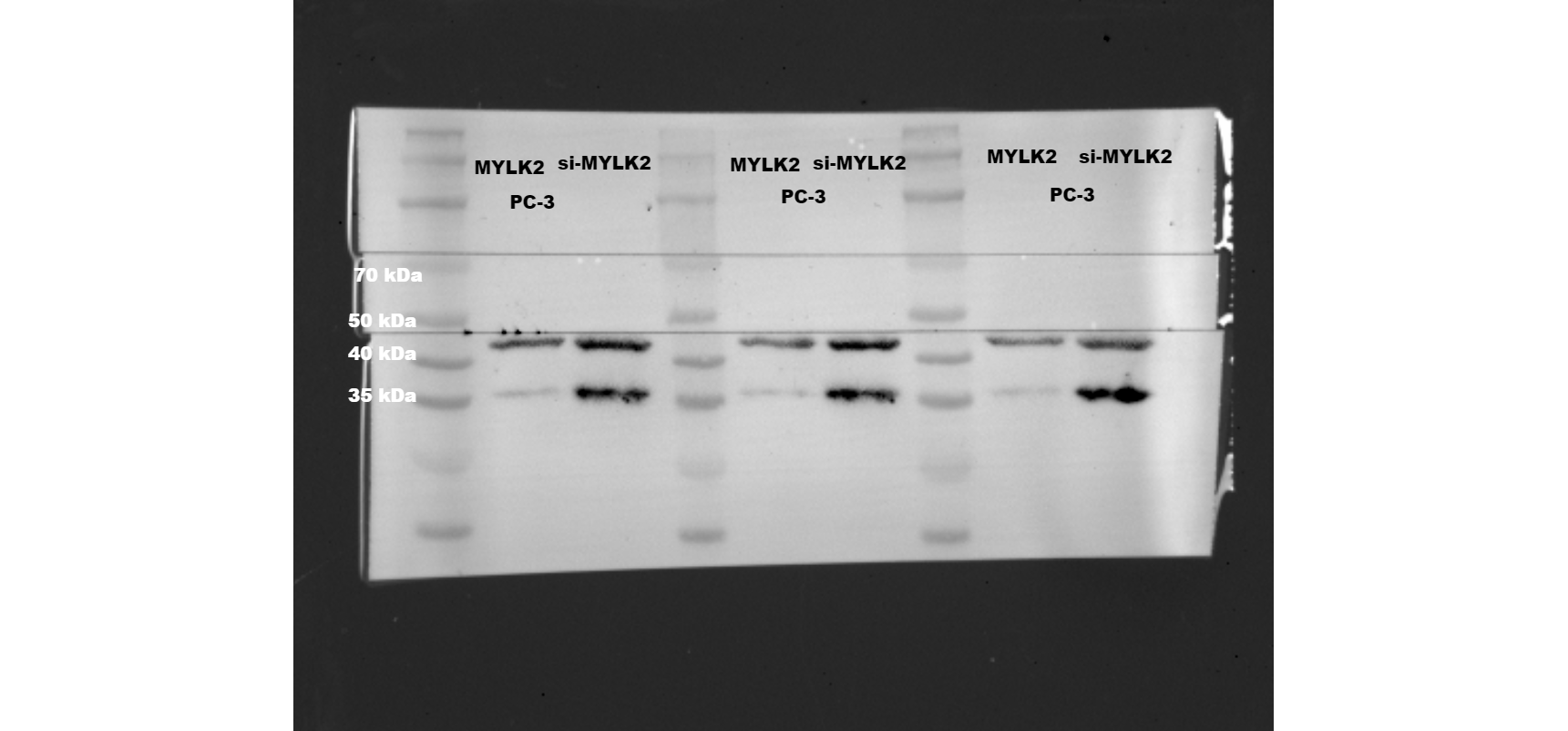

Supplement: Supplementary file 3 [file Image11.png]

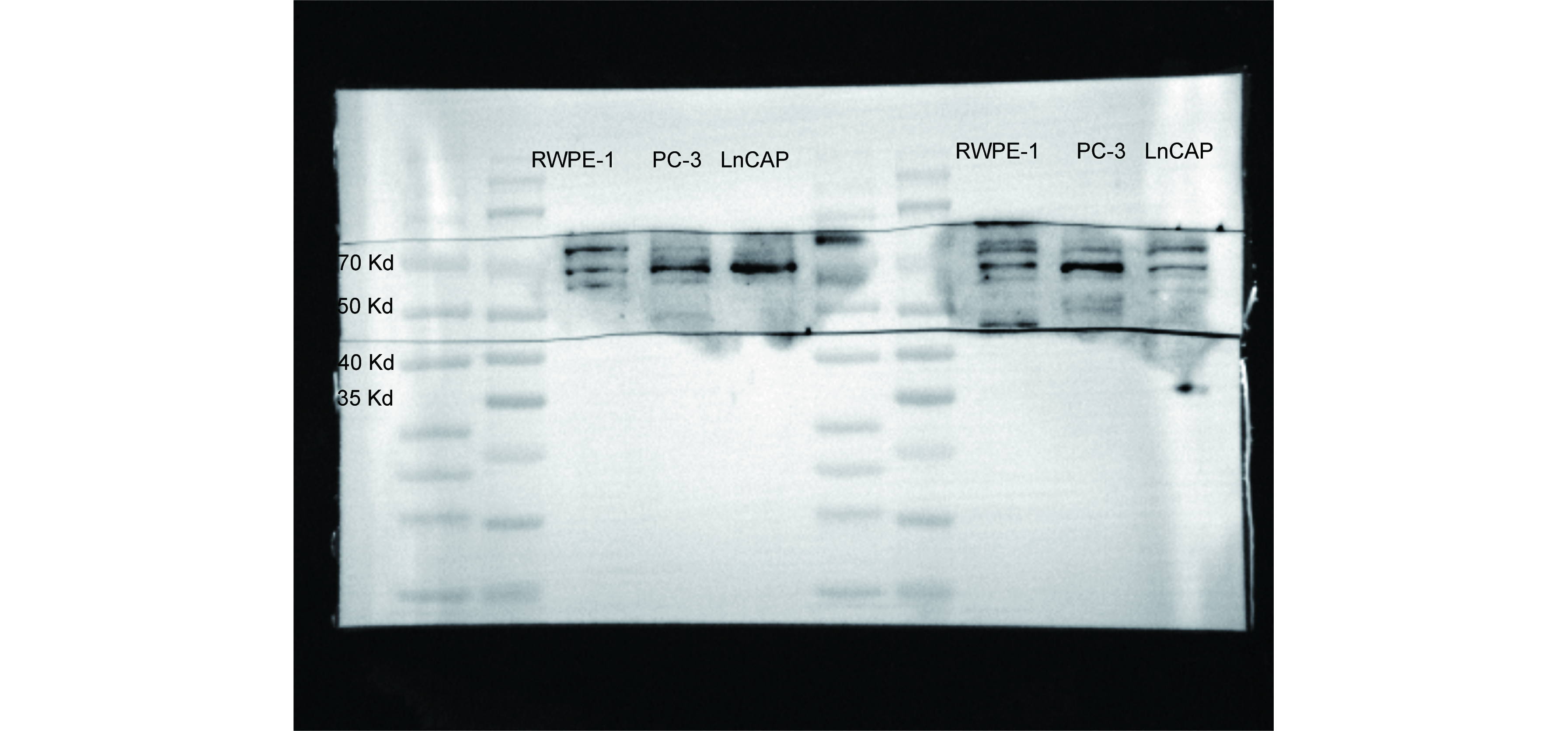

Supplement: Supplementary file 4 [file Image3.tif]

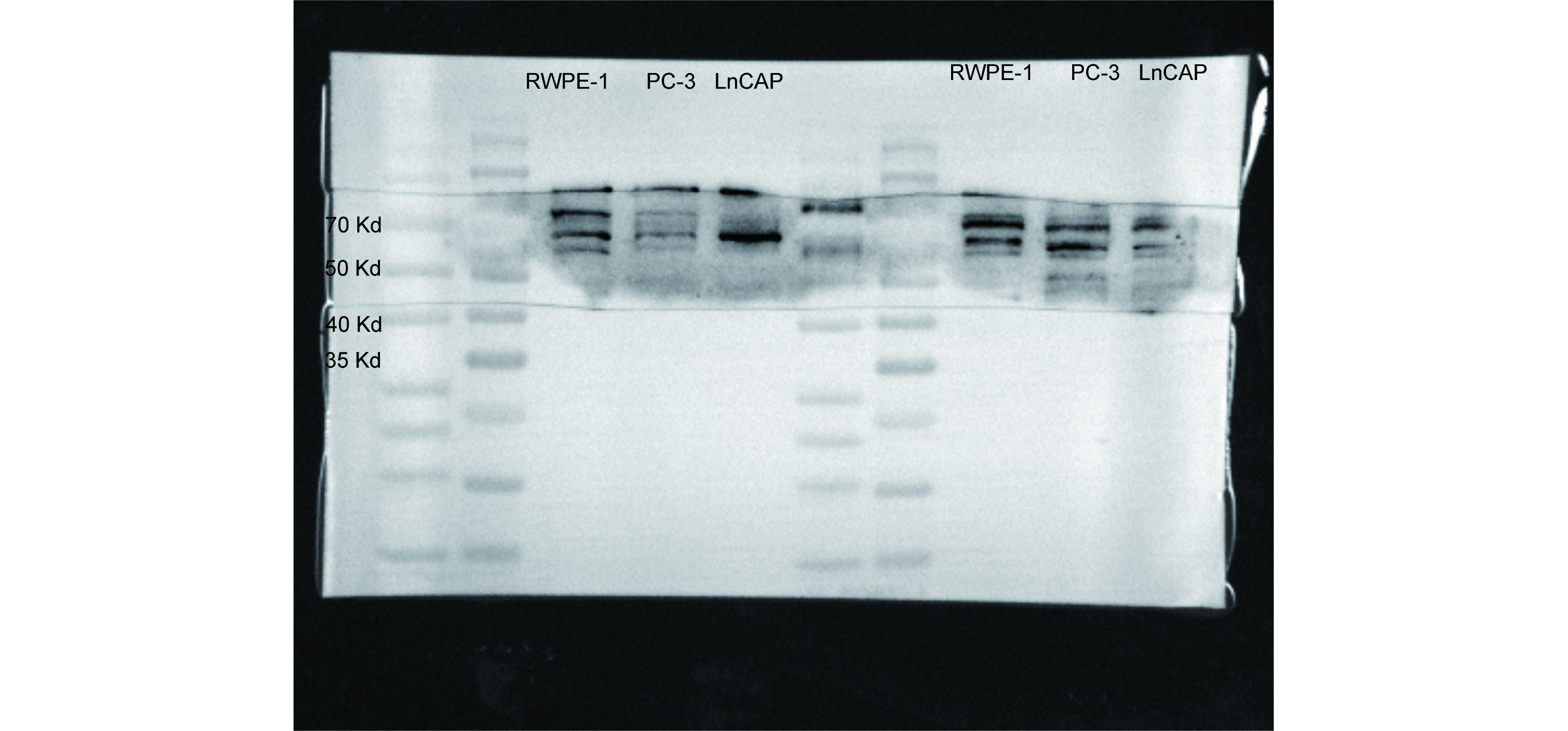

Supplement: Supplementary file 5 [file Image4.tif]

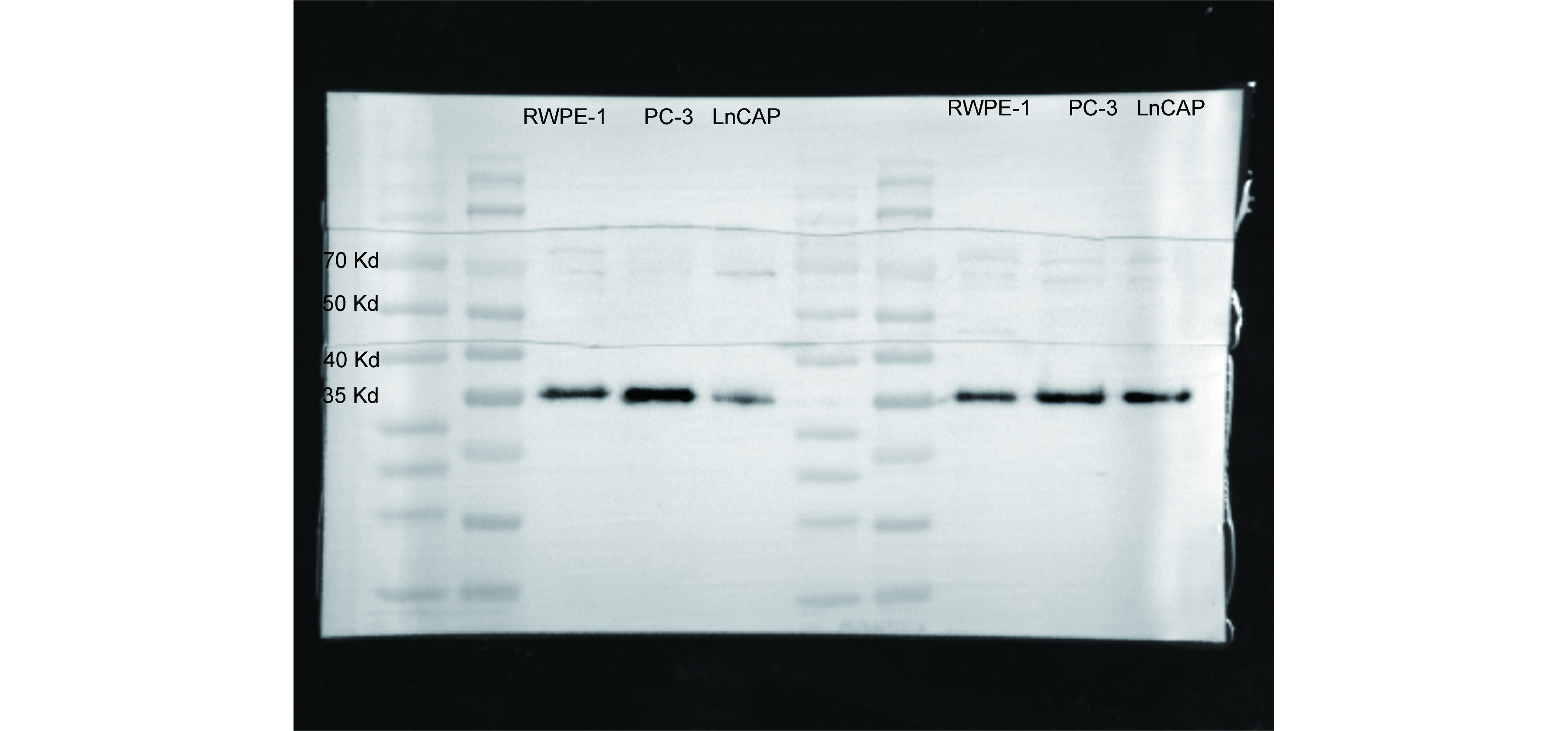

Supplement: Supplementary file 6 [file Image2.tif]

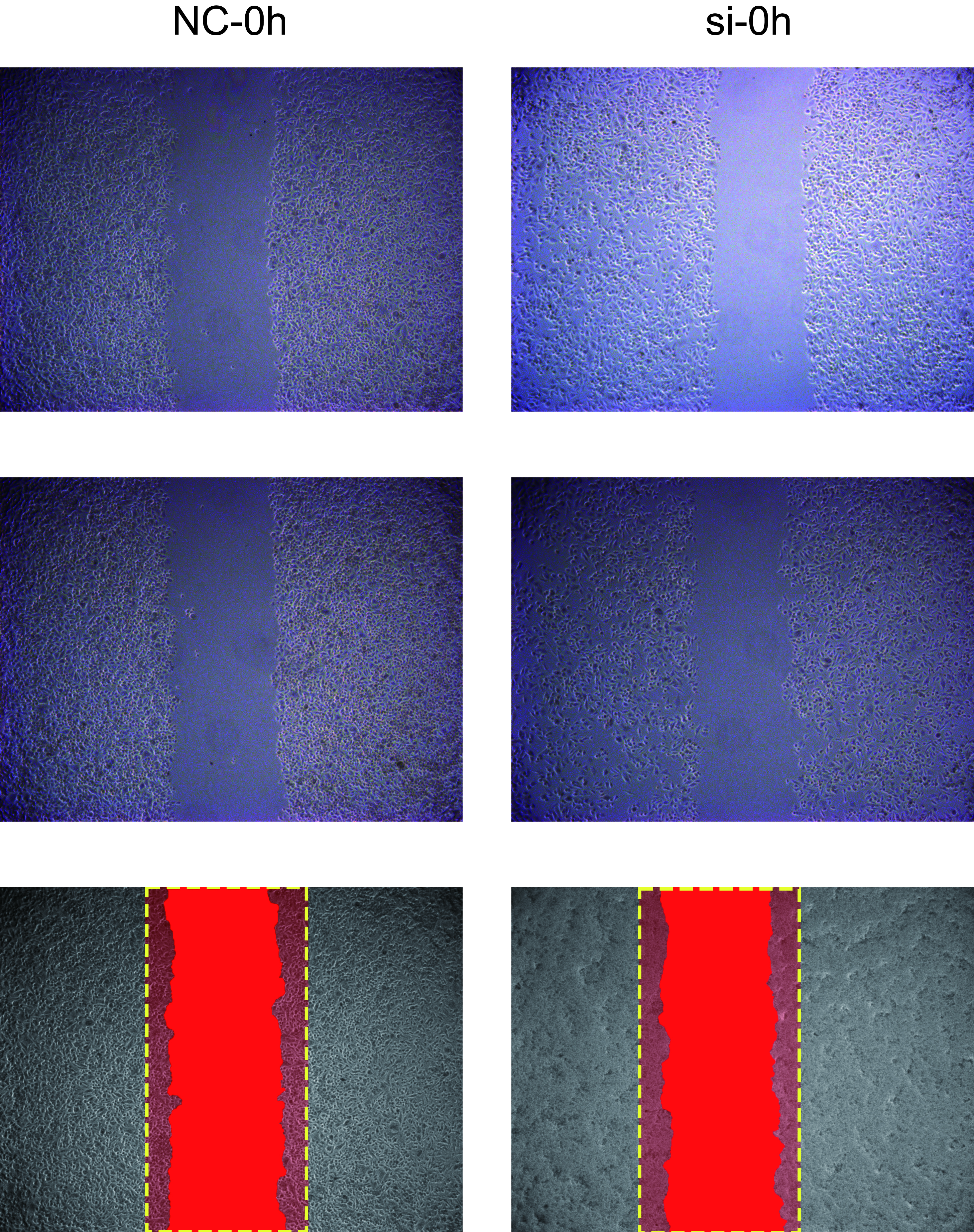

Supplement: Supplementary file 8 [file Image5.jpeg]

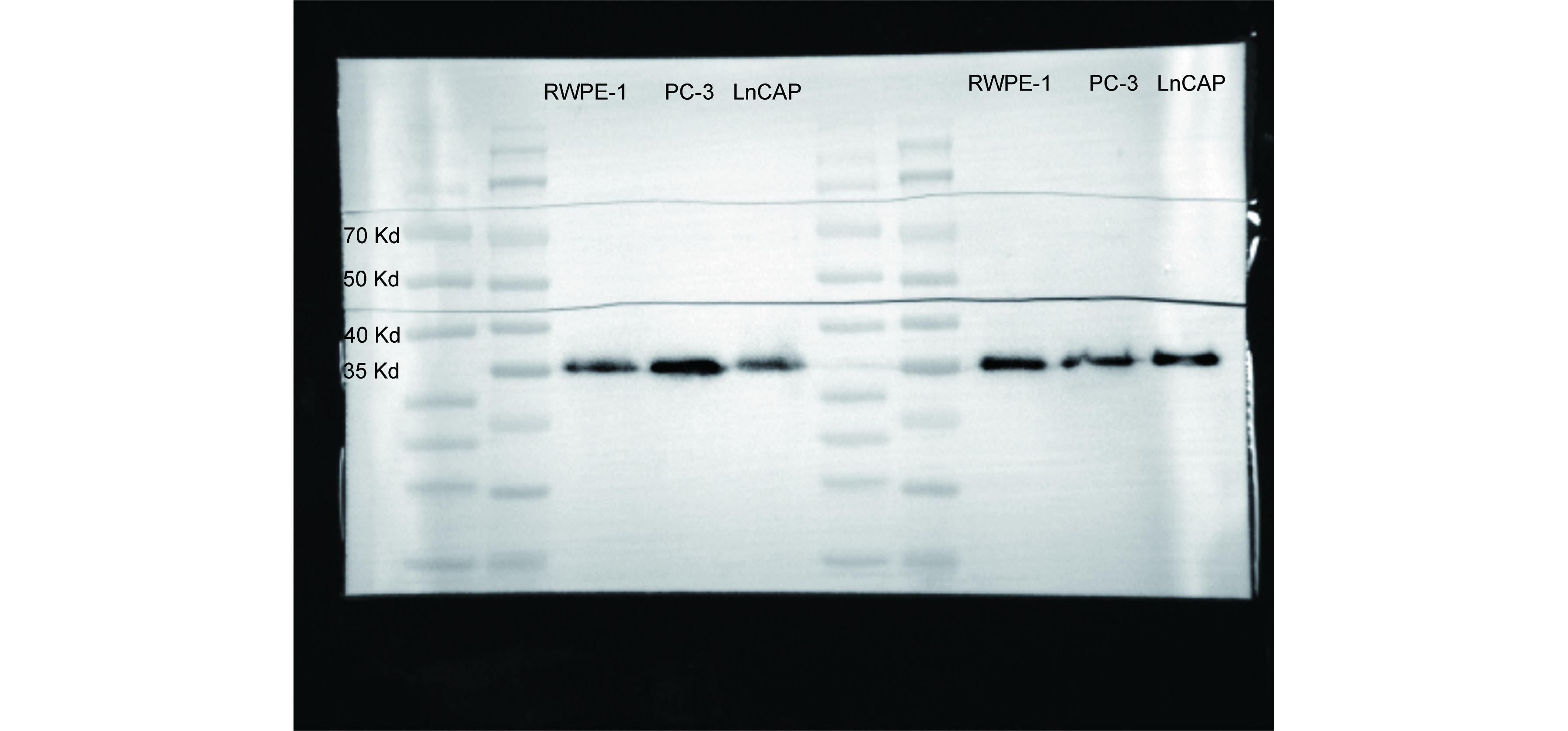

Supplement: Supplementary file 9 [file Image1.tif]

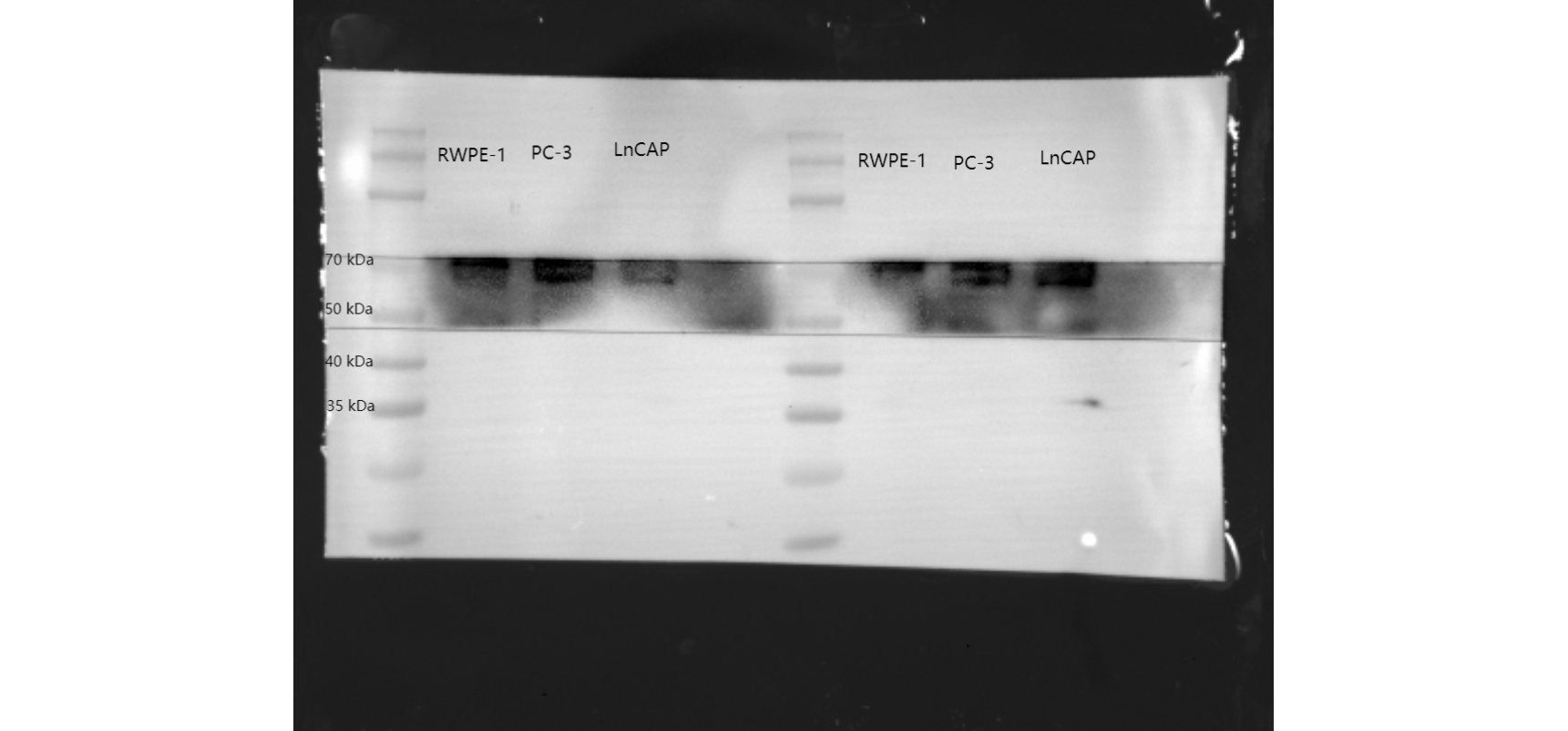

Supplement: Supplementary file 12 [file Image8.png]

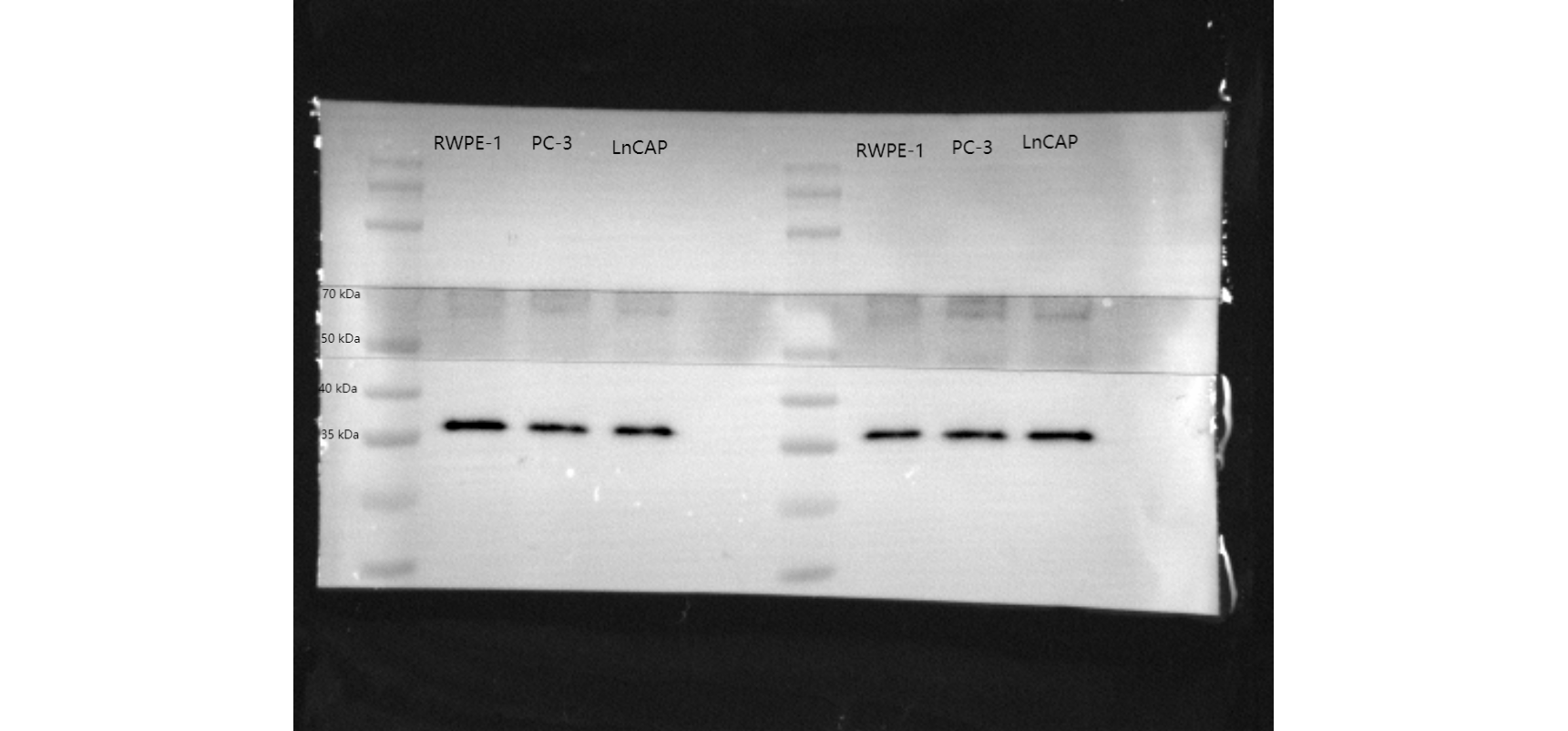

Supplement: Supplementary file 13 [file Image9.png]

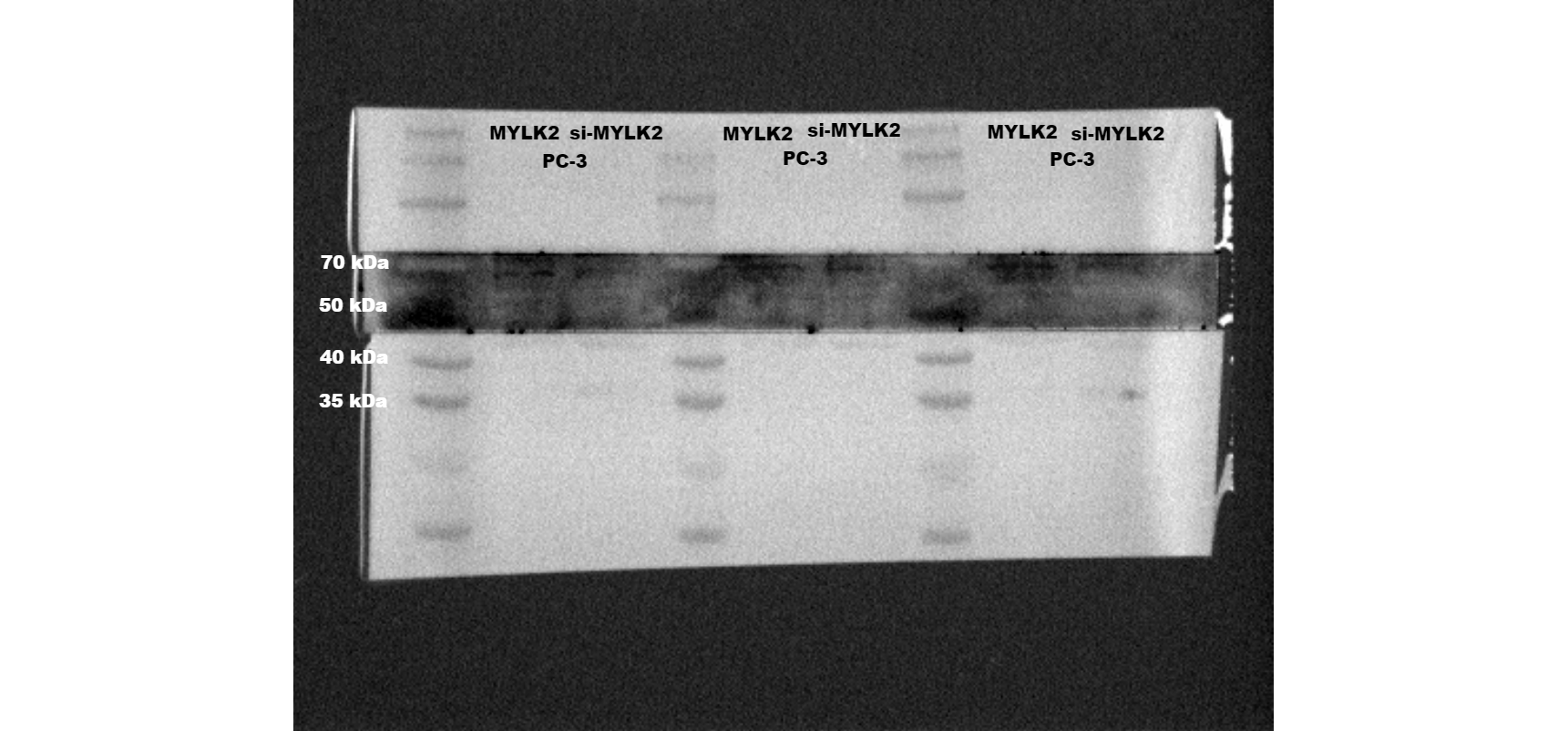

Supplement: Supplementary file 14 [file Image10.png]

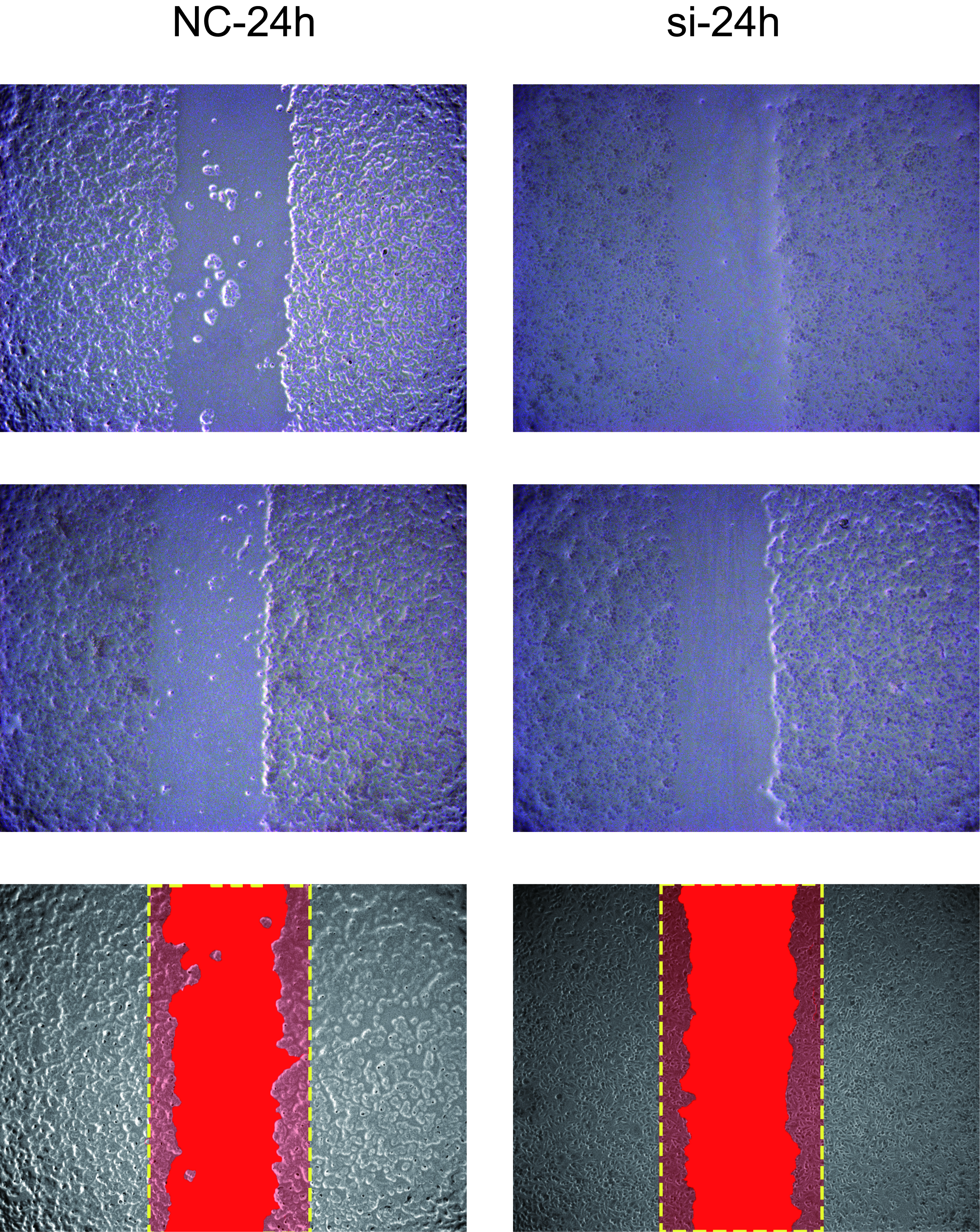

Supplement: Supplementary file 15 [file Image6.jpeg]
