# Supplementary material for: Impact of ITH on PRAD patients and feasibility analysis of the positive correlation gene MYLK2 applied to PRAD treatment
Source: Front Genet. 2025 May 20;16:1589259. doi: 10.3389/fgene.2025.1589259 (PMC12130005; doi:10.3389/fgene.2025.1589259)
Supplement: Supplementary file 10 [file DataSheet1.pdf]

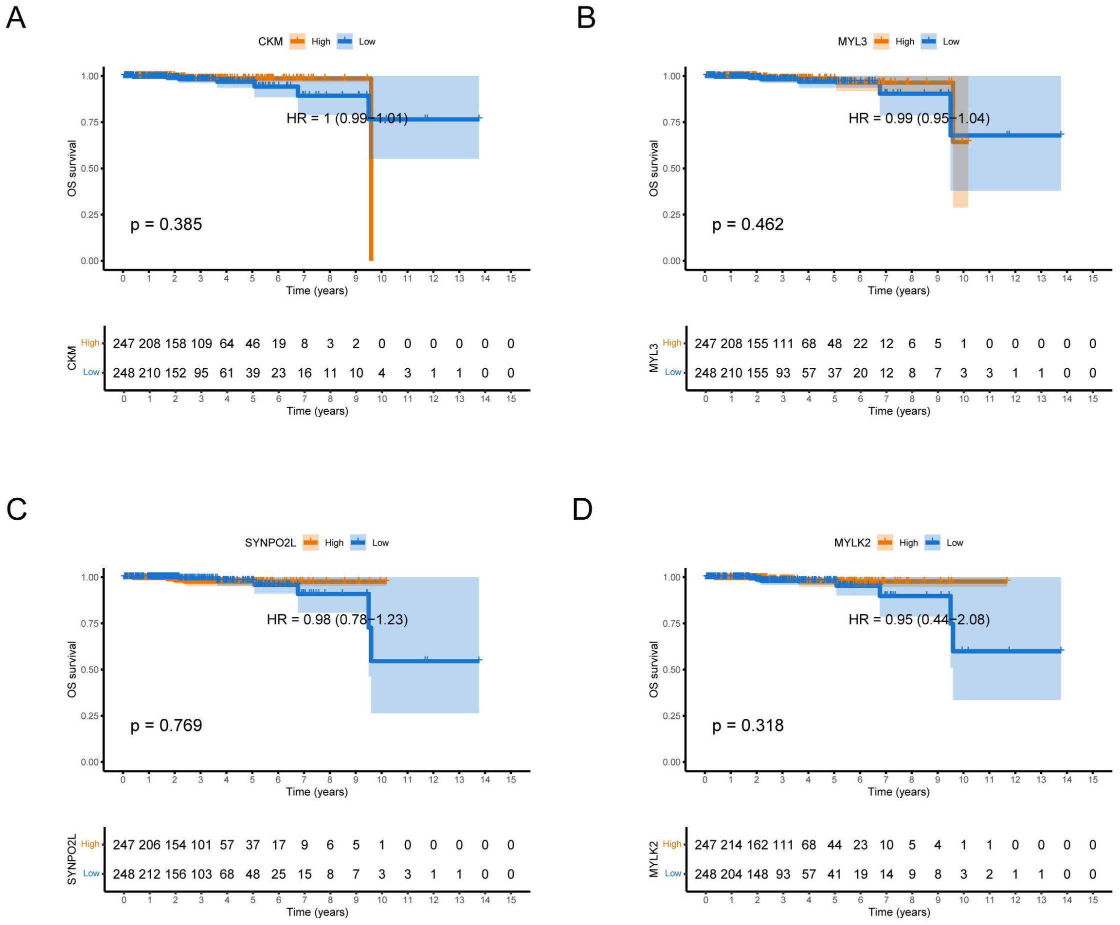

Figure S1 (A-D)The overall survival curve of ITH high correlation genes CKM, MYL3, SYNPO2L and MYLK2.

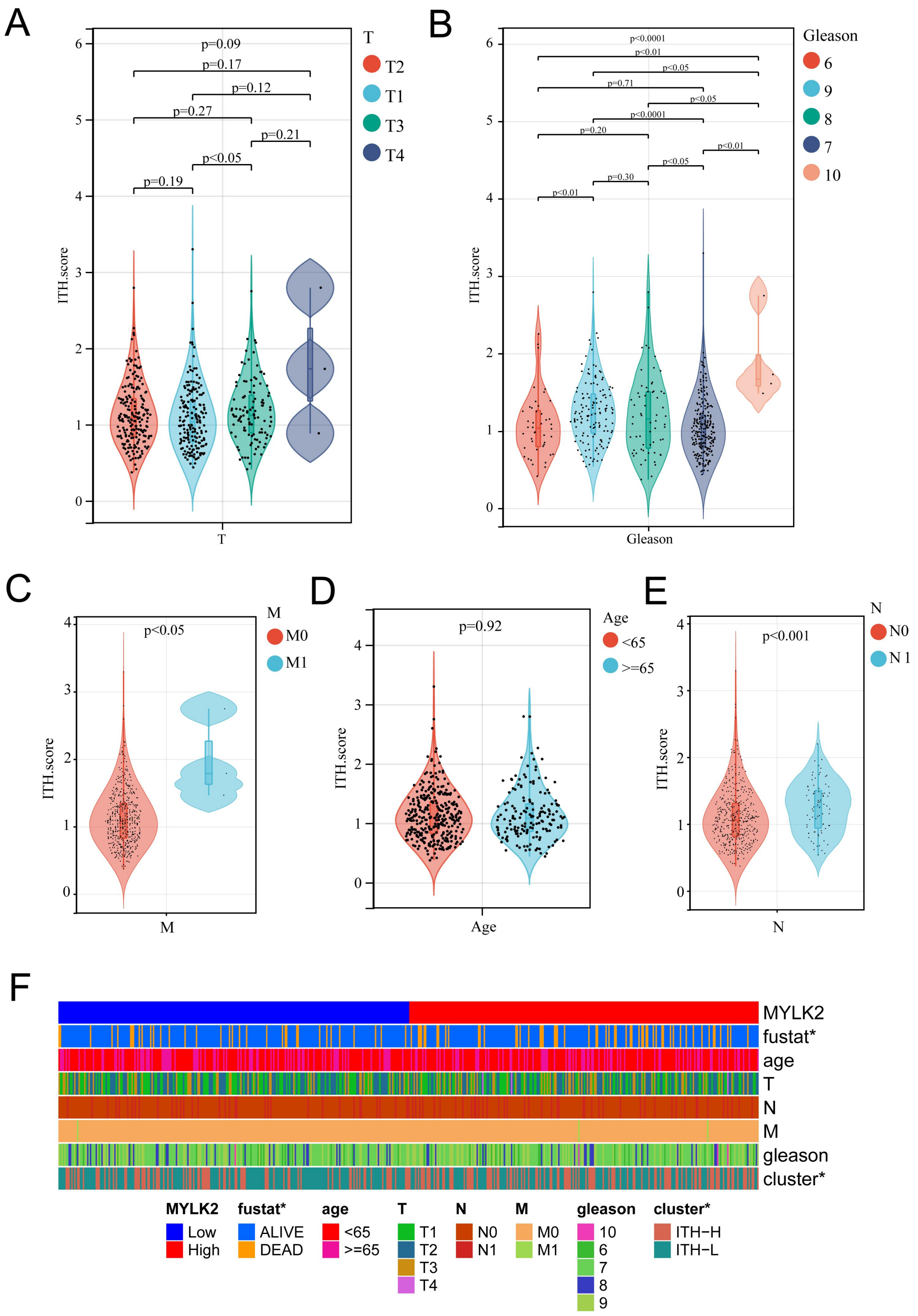

Figure S2 (A) The difference of ITH-score levels in different T stages. (B) Differences in ITH-score levels under different Gleason scores. (C) The difference of ITH-score levels in different M stages. (D) Differences in ITH-score levels under different ages. (E) The difference of ITH-score levels in different N stages. (F) The heat map summarizes the correlation between all the above clinical information and ITH-score.

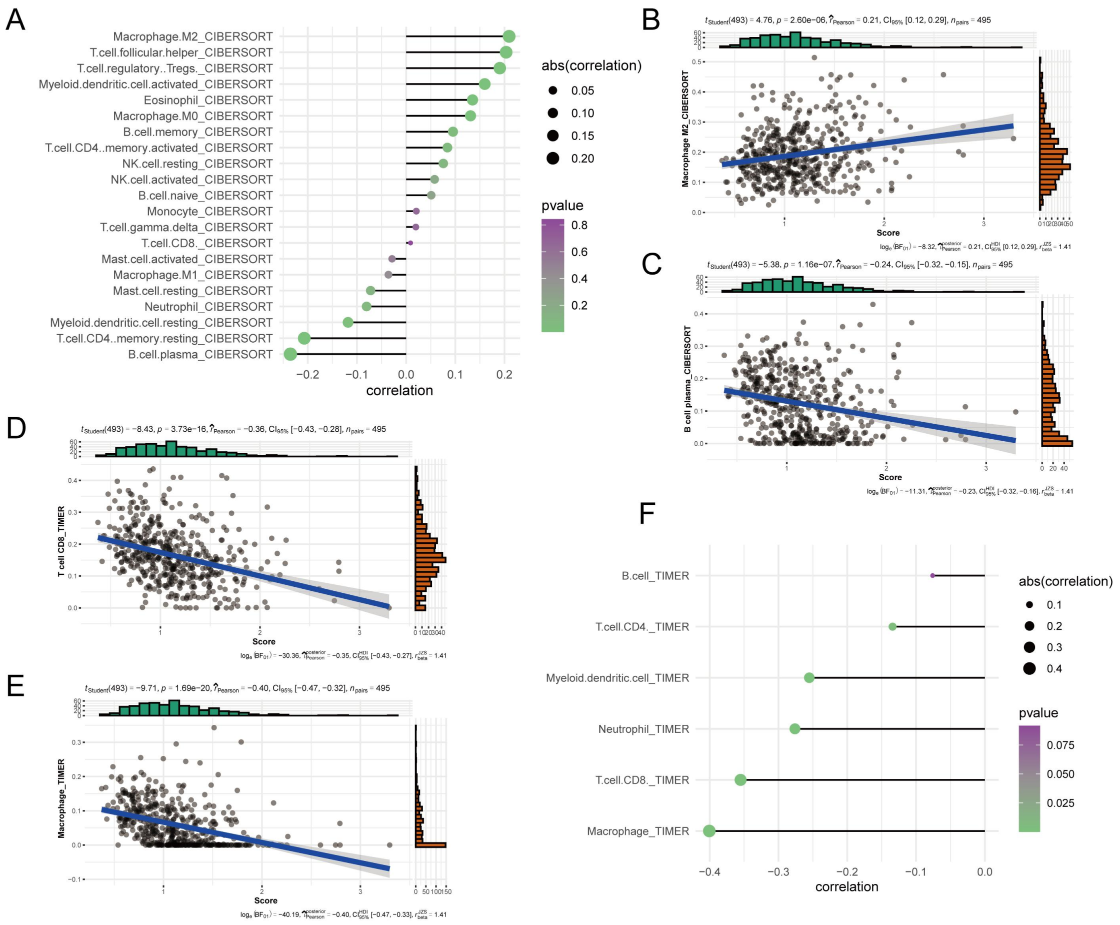

Figure S3 (A-C) The correlation between each immune infiltration component and ITH-score under CIBERSORT algorithm. (D-F) The correlation between each immune infiltration component and ITH-score under TIMER algorithm.

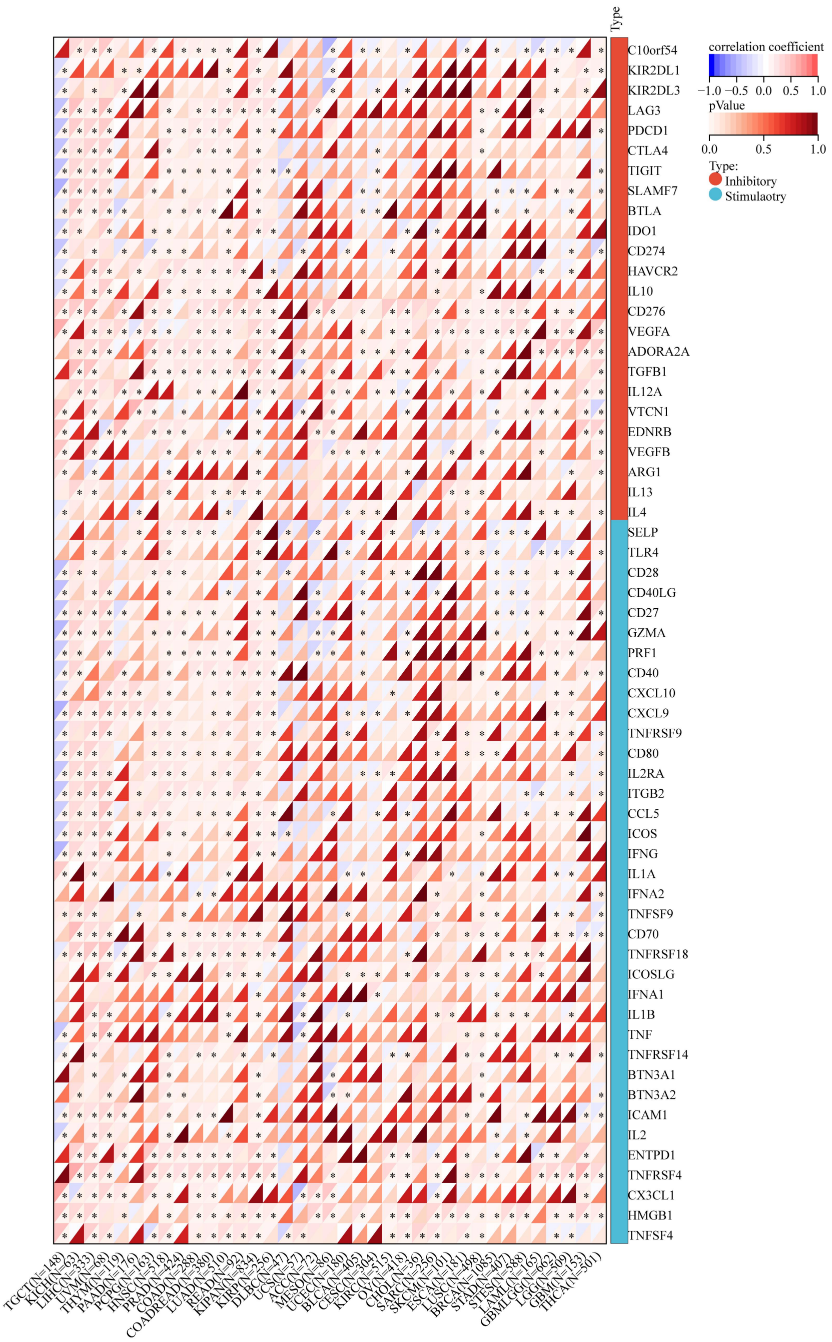

Figure S4 The heat map shows the results of pan-cancer analysis: the correlation between ITH-score and immune checkpoint key genes under different cancer types.

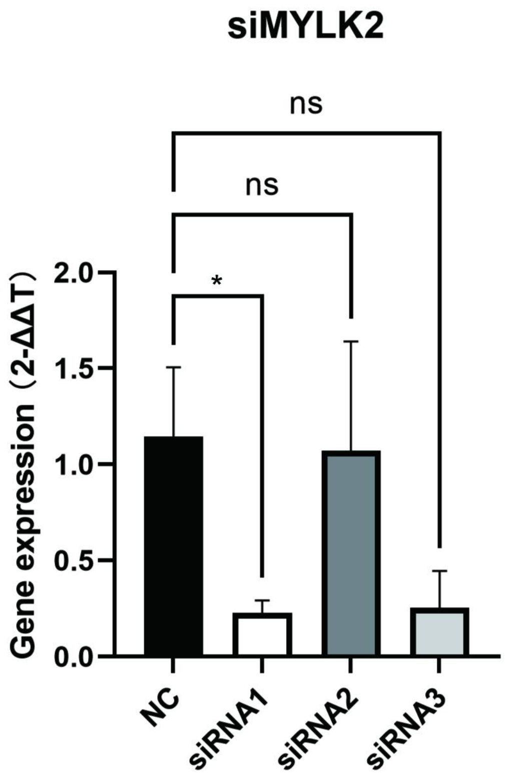

Figure S5 The siMYLK2 validation by qPCR.

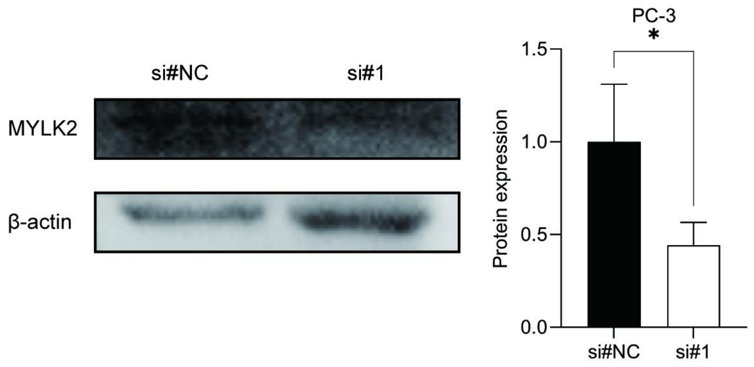

Figure S6 siMYLK2 validation by western blot .
